# Predicting Adverse Cardiac Events After Radiotherapy for Locally Advanced Non–Small Cell Lung Cancer

**DOI:** 10.1016/j.jaccao.2023.08.007

**Published:** 2023-10-04

**Authors:** Hyunsoo Joshua No, Felicia B. Guo, Natalie Jung-In Park, Noah Kastelowitz, June-Wha Rhee, Daniel Eugene Clark, Alexander Li-Che Chin, Lucas Kas Vitzthum, Kathleen Claire Horst, Everett James Moding, Billy W. Loo, Maximilian Diehn, Michael Sargent Binkley

**Affiliations:** a Department of Radiation Oncology, Stanford University School of Medicine, Stanford, California, USA; b University of Rochester School of Medicine and Dentistry, Rochester, New York, USA; c Department of Medicine, Division of Cardiology, City of Hope Comprehensive Cancer Center, Duarte, California, USA; d Division of Cardiovascular Medicine, Department of Medicine, Stanford University School of Medicine, Stanford, California, USA

**Keywords:** cardiac substructures, cardiotoxicity, coronary artery calcium, non–small cell lung cancer, radiotherapy

## Abstract

**Background:**

Radiotherapy may cause grade ≥3 cardiac events, necessitating a better understanding of risk factors. The potential predictive role of imaging biomarkers with radiotherapy doses for cardiac event occurrence has not been studied.

**Objectives:**

The aim of this study was to establish the associations between cardiac substructure dose and coronary artery calcium (CAC) scores and cardiac event occurrence.

**Methods:**

A retrospective cohort analysis included patients with locally advanced non–small cell lung cancer treated with radiotherapy (2006-2018). Cardiac substructures, including the left anterior descending coronary artery, left main coronary artery, left circumflex coronary artery, right coronary artery, and TotalLeft (left anterior descending, left main, and left circumflex coronary arteries), were contoured. Doses were measured in 2-Gy equivalent units, and visual CAC scoring was compared with automated scoring. Grade ≥3 adverse cardiac events were recorded. Time-dependent receiver-operating characteristic modeling, the log-rank statistic, and competing-risk models were used to measure prediction performance, threshold modeling, and the cumulative incidence of cardiac events, respectively.

**Results:**

Of the 233 eligible patients, 61.4% were men, with a median age of 68.1 years (range: 34.9-90.7 years). The median follow-up period was 73.7 months (range: 1.6-153.9 months). Following radiotherapy, 22.3% experienced cardiac events, within a median time of 21.5 months (range: 1.7-118.9 months). Visual CAC scoring showed significant correlation with automated scoring (*r* = 0.72; *P* < 0.001). In a competing-risk multivariable model, TotalLeft volume receiving 15 Gy (per 1 cc; HR: 1.38; 95% CI: 1.11-1.72; *P* = 0.004) and CAC score >5 (HR: 2.51; 95% CI: 1.08-5.86; *P* = 0.033) were independently associated with cardiac events. A model incorporating age, TotalLeft CAC (score >5), and volume receiving 15 Gy demonstrated a higher incidence of cardiac events for a high-risk group (28.9%) compared with a low-risk group (6.9%) (*P* < 0.001).

**Conclusions:**

Adverse cardiac events associated with radiation occur in more than 20% of patients undergoing thoracic radiotherapy within a median time of <2 years. The present findings provide further evidence to support significant associations between TotalLeft radiotherapy dose and cardiac events and define CAC as a predictive risk factor.

The leading noncancer cause of morbidity and mortality among long-term cancer survivors is cardiovascular (CV) disease. Those who undergo thoracic radiotherapy (RT) exhibit increased rates of grade 3 or higher^1^ adverse cardiac events in comparison with nonirradiated survivors, as highlighted by previous studies.^2^, ^3^, ^4^ Furthermore, more than 10% of patients with lung cancer may experience such cardiac events within 2 years of receiving RT.^3^ Thus, minimizing cardiotoxicity after thoracic RT remains a substantial clinical challenge, underscoring the importance of identifying predictive factors associated with these outcomes.

The mean RT doses received by the heart have been associated with cardiotoxicity,^4^, ^5^, ^6^, ^7^, ^8^ with an estimated 4% to 16% increased risk for cardiac events per 1 Gy received. However, it has been suggested that total heart dose metrics may serve as a surrogate for damage to specific cardiac substructures.^9^ Recent studies suggest that RT doses to coronary arteries and cardiac substructures could offer better quantification of risk after RT,^10^^,^^11^ providing more specific metrics for assessing cardiotoxicity. There is emerging interest in investigating RT doses to cardiac substructures, such as the coronary arteries, as a means to better quantify the risk for cardiac events after RT. This approach can provide valuable insights for guiding future treatment strategies aimed at reducing CV risk.

Additionally, coronary artery calcium (CAC) score, a direct imaging measure of atherosclerotic burden, has been reported as a highly specific imaging biomarker for CV risk,^12^^,^^13^ and CAC score may provide additional cardiotoxicity risk stratification after RT.^14^^,^^15^ CAC scoring has shown a strong predictive capability for future CV risk, and the extent of pre-RT CAC burden has demonstrated an independent association with cardiac events after RT, even when adjusting for mean heart dose (MHD).^16^ Although comprehensive automated Agatston scoring is available, simple visual assessment of CAC scoring can generate risk assessments for CV-related death and all-cause mortality, showing a strong association with outcomes.^17^

We sought to enhance our understanding of predictive factors associated with higher risks for future adverse cardiac events following thoracic RT by combining clinical, dosimetric, and CAC risk factors. Furthermore, we investigated the potential of an integrated model to improve the prediction of adverse cardiac events after thoracic RT. This approach aims to provide management solutions for mitigating long-term CV risks among cancer survivors.

## Methods

### Clinical data

A retrospective review was performed with Institutional Review Board approval provided by the Stanford Research Compliance Office on consecutively treated patients with locally advanced non–small cell lung cancer (NSCLC) who received RT as their primary treatment at our institution from 2006 through 2018. All patients were diagnosed with stage IIB to IIIC NSCLC.^18^ Baseline cardiac risk, including pretreatment cardiac history and data from the American College of Cardiology atherosclerotic CV disease (ASCVD) risk estimator,^19^ was recorded. This risk estimator incorporates age, sex, race, smoking history, diabetes status, medications, blood pressure, and cholesterol levels in those with available data. Blood pressure readings were obtained from the initial radiation oncology consultation, and cholesterol levels were obtained up to 1 year prior to the initial consultation date. Treatment details including radiation dose, fractionation, RT technique, prior RT, and chemotherapy regimen (before, during, or after RT) were extracted from hospital records.

### Classifying grade ≥3 adverse cardiac events

Classifying grade ≥3 adverse cardiac events involved categorizing them into 4 distinct types: myocardial, constrictive, valvular, and conduction. Myocardial events included conditions such as heart failure with preserved or reduced ejection fraction, new coronary artery disease, unstable angina, myocardial infarction, and other ischemic events. Constrictive events referred to pericarditis. Valvular events involved any form of valve-related disease. Conduction events included abnormalities requiring a permanent pacemaker or second- or third-degree heart block, as well as newly occurring atrial arrhythmia or ventricular arrhythmia requiring interventions.

### Dosimetric analysis

**Figure 1:**
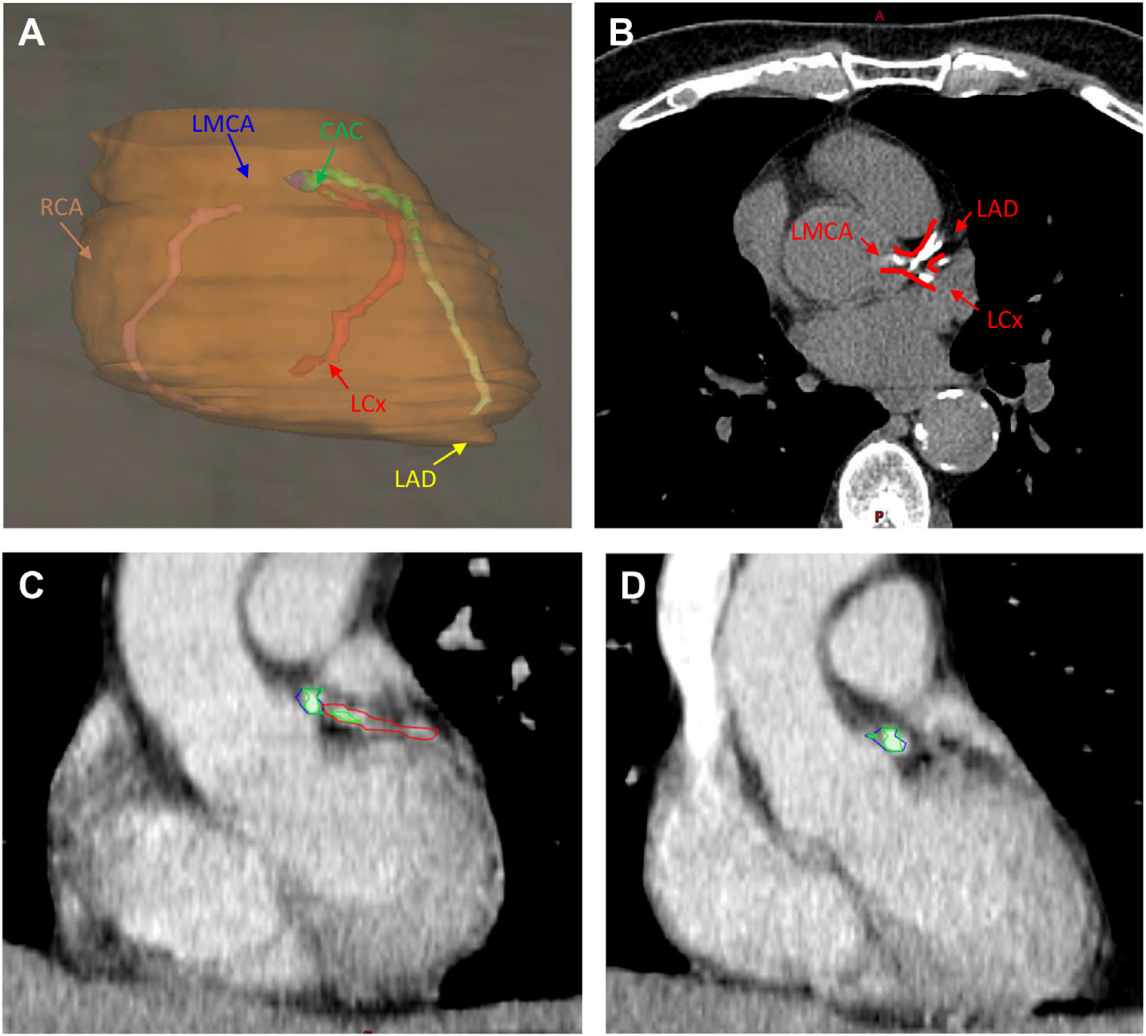
Visual Scoring of CAC (A) Three-dimensional rendering of the whole heart (orange), left main coronary artery (LMCA) (blue), left anterior descending coronary artery (LAD) (yellow), left circumflex coronary artery (LCx) (red), and right coronary artery (RCA) (pink). Coronary artery calcium (CAC) (green) was scored on the basis of visual assessment of vessel involvement. (B) CAC scoring performed by evaluating high-density calcifications throughout the lengths of individual affected vessels. (C) Mild vessel involvement, with small volume CAC (green) involving less than one-third of the LCx (red) was scored 1. Intermediate involvement between 1 and 3 was scored 2, as seen in the LAD in (A). (D) High vessel involvement with full CAC (green) involvement of the LMCA (blue) was scored 3.

The RT plans were transferred to MIM Radiation Oncology (MIM Software). All doses were converted to equivalent doses in 2-Gy fractions using an α/β ratio of 3 for organs at risk. In cases in which patients underwent multiple courses of thoracic RT, plans from separate computed tomographic (CT) simulation scans were fused on the initial thoracic treatment CT study. Plans not involving the thorax were excluded from the analysis. Subsequently, the heart, left ventricle (LV), left anterior descending coronary artery (LAD), left main coronary artery (LMCA), left circumflex coronary artery (LCx), and right coronary artery (RCA) were contoured on the primary CT data set (Figure 1A) by a single blinded observer (H.J.N.). A 5-mm brush was used to contour the individual cardiac vessels, guided by a cardiac contouring atlas.^20^ The following metrics were tabulated: mean doses received by the heart, LV, LAD, LMCA, LCx, RCA, combined 3-vessel arteries (including the LAD, LMCA, and LCx, referred to as TotalLeft), and all combined coronary arteries (referred to as TotalCor), as well as the volume receiving 15 Gy (V15) of the heart, LV, LAD, LMCA, LCx, RCA, TotalLeft, and TotalCor. In alignment with findings from Atkins et al,^10^ we also measured the volume receiving 27 Gy of the LMCA and the volume receiving 7 Gy of the TotalCor structure. All volume metrics were obtained as both percentages of the structure and total cubic centimeters, reflecting the dose of interest. Dosimetric analysis was performed for those with and without multiple courses of RT, as well as across various RT techniques.

### CAC scoring

CAC scores were tabulated using a modified Chiles method,^17^ evaluating the initial treatment simulation CT study for all patients.^21^ Although the initial intention was to use automated Agatston scoring, a significant portion of images were obtained with intravenous contrast, rendering automated Agatston methods suboptimal for scoring. Thus, CAC scoring was performed through visual analysis of individual coronary vessels (Figure 1B). For scoring, absent CAC was assigned a score of 0, mild CAC a score of 1 (Figure 1C), moderate CAC a score of 2, and heavy CAC a score of 3 for each epicardial vessel (Figure 1D). All images were standardized to a preset mean and window of −125 to 225 HU. All vessels were visually segmented into 3 equal portions for evaluation, focusing on the site with the most obvious CAC involvement. CAC was categorized as mild if only isolated flecks were present within a single segment (one-third of the vessel). Moderate classification was assigned if calcium deposits were present in 2 segments (two-thirds of the vessel). Heavy classification was assigned if continuous CAC was observed within 3 segments (the entire vessel). CAC identification was carried out by a single trained technician (H.J.N.) and verified by a fellowship-trained cardiac radiologist (D.E.C.), who was blinded to clinical information. For images with intravenous contrast, a 300-HU cutoff was used to differentiate between contrast media and calcifications.^22^^,^^23^

Visual CAC scoring was compared with established automated Agatston scoring using AVIEW CAC (Coreline Soft), a U.S. Food and Drug Administration–approved automated CAC scoring system. Thirty cases with visually scored mild, moderate, and severe classifications, equally distributed, and involving noncontrast imaging underwent automated Agatston scoring to enable a comparison.

### Statistical analysis

Follow-up was calculated using the reverse Kaplan-Meier method, with day 0 being the first day of RT. Model performance for dose and cardiac calcification metric was assessed using receiver-operating characteristic (ROC) curves, using a competing risk ROC method (with the R package timeROC). To assess the prediction performance of the cardiac substructure mean dose for cardiac events, time-dependent ROC modeling at 5 years post-RT was performed. For threshold modeling, we used the log-rank statistic, with 95% CIs measured using bootstrap resampling with 1,000 iterations. Competing-risk models were constructed using Fine-Gray subdistribution hazard models. Competing-risk models, considering death as the competing risk, were used to measure the cumulative incidence of cardiac events. These models were used in both univariable and multivariable risk regression modeling, with the results presented as HRs and 95% CIs. For the competing-risk regression models, we visually assessed the proportional hazards assumption using log(−log[survival]) plots. In general, variables with *P* values ≤0.05 in univariable analyses were included in multivariable models. For significant collinear dose variables, we included the variables with the highest areas under the curve (AUCs) from our ROC analyses, which included age, TotalLeft V15, and TotalLeft CAC score. Pearson correlation coefficient analysis was used to validate CAC scoring. All analyses were performed using R version 4.2.2 (R Foundation for Statistical Computing). A 2-sided *P* value of <0.05 was considered to indicate statistical significance.

## Results

We identified 233 consecutive patients with locally advanced NSCLC who underwent primary RT, with or without chemotherapy. The median age was 68.1 years (range: 34.9-90.7 years), and 61.4% (n = 143) were men (Table 1). The median follow-up duration was 73.7 months (range: 1.6-153.9 months), and the median overall survival was 34.8 months (Supplemental Figure 1). Among all patients, 81.1% (n = 189) reported smoking histories, while 12.9% (n = 30) reported actively smoking at the initiation of RT. Comorbidities in the cohort included diabetes diagnosed in 22.3% of patients (n = 52), hypertension in 51.9% (n = 121), and hypercholesterolemia with active statin use in 39.9% (n = 93). Only 18.9% (n = 44) had sufficient laboratory information to calculate ASCVD scores, with a median 10-year ASCVD risk of 21.5% (range: 0.4%-71.1%). Nearly all patients (97.9% [n = 228]) received concurrent chemotherapy with RT, and 15.4% (n = 36) received neoadjuvant courses. Additionally, 36.1% (n = 84) received at least 1 chemotherapy cycle following their initial course of RT. Among them, 57 (24.5%) received multiple courses of thoracic RT, without experiencing any cardiac events prior to the delivery of subsequent RT courses. Comparing cardiac event outcomes, there was no significant difference observed between those who underwent multiple RT courses and those who did not (HR: 0.43; 95% CI: 0.15-1.20; *P* = 0.11) (Table 2). Regarding RT technique, 74.7% (n = 174) underwent volumetric modulated arc therapy, followed by 24.9% (n = 58) who received static-angle intensity modulated RT, and 0.4% (n = 1) who received 3-dimensional conformal RT (Table 1). Comparing adverse cardiac event outcomes, no significant difference was found between those who received volumetric modulated arc therapy and those who did not (HR: 0.43; 95% CI: 0.15-1.25; *P* = 0.12) (Table 2).

**Table 1:** Baseline Patient Characteristics (N = 233)

Follow-up, mo	73.7 (1.6-153.9)
Age at radiotherapy initiation, y	68.1 (34.9-90.7)
Male	143 (61.4)
Ever smoker	189 (81.1)
Current smoker	30 (12.9)
Diabetes	52 (22.3)
Hypertension	121 (51.9)
Statin use	93 (39.9)
Median 10-y ASCVD risk, %^a^	21.5 (0.4-71.1)
Concurrent chemotherapy	228 (97.9)
Prior chemotherapy	36 (15.4)
Postradiotherapy chemotherapy	84 (36.1)
Multiple courses of thoracic radiotherapy	38 (16.3)
Radiotherapy technique	
VMAT	174 (74.7)
IMRT	58 (24.9)
3D CRT	1 (0.4)
Prior cardiac events	71 (30.5)
Multiple prior cardiac events	35 (15.0)
Grade ≥3 cardiac events following radiotherapy	52 (22.3)
Myocardial	19 (36.5)
Constrictive	3 (5.8)
Valvular	3 (5.8)
Conduction	27 (51.9)
Time to first postradiotherapy cardiac event, mo	21.5 (1.7-118.9)
Cohort heart dosimetry	
Mean heart dose, Gy	9.3 (IQR: 2.5-12.9)
Mean TotalLeft V15, %	21.6 (IQR: 0.0-37.8)
Mean TotalLeft V15, cc	1.2 (IQR: 0.0-2.1)
CAC score distribution	
None	52 (22.3)
Mild	62 (26.6)
Moderate	58 (24.9)
Severe	61 (26.2)

Values are median (range) or n (%) except as indicated.

Chemotherapy regimens listed in Supplemental Table 3.

3D = 3-dimensional; ASCVD = atherosclerotic cardiovascular disease; CAC = coronary artery calcium; CRT = conformal radiotherapy; IMRT = intensity-modulated radiotherapy; TotalLeft = combined 3-vessel arteries; V15 = volume receiving 15 Gy; VMAT = volumetric modulated arc therapy.

a Clinical and laboratory information was sufficient to calculate ASCVD score for 18.9% of the total cohort (n = 44).

**Table 2:** Univariable and Multivariable Models for Grade ≥ 3 Cardiac Events Considering Noncardiac Death as a Competing Risk

	Univariable			Multivariable		
	HR	95% CI	*P* Value	HR	95% CI	*P* Value
Age	1.04	1.01-1.07	0.012	1.03	0.99-1.06	0.13
Male	1.53	0.70-3.33	0.29			
Ever smoker	3.65	0.89-15.1	0.073			
Diabetes	1.32	0.59-2.95	0.50			
Hypertension	1.26	0.61-2.59	0.53			
Statin	1.80	0.88-3.68	0.11			
MHD, per 5 Gy	1.12	1.09-1.16	<0.001			
LCx volume receiving 15 Gy, per 1 cc	1.69	1.20-2.39	0.003			
RCA volume receiving 15 Gy, per 1 cc	1.26	0.76-2.09	0.38			
LAD volume receiving 15 Gy, per 1 cc	1.88	1.17-3.02	0.009			
LMCA volume receiving 15 Gy, per 1 cc	1.67	0.44-6.29	0.45			
TotalLeft volume receiving 15 Gy, per 1 cc	1.34	1.08-1.66	0.007	1.38	1.11-1.72	0.004
TotalCor volume receiving 7 Gy, per 1 cc	1.16	0.99-1.36	0.068			
TotalLeft calcium score, per 1 point	1.19	1.04-1.37	0.011			
TotalLeft calcium score >5 points	2.96	1.43-6.15	0.004	2.51	1.08-5.86	0.033
Cardiac history	1.63	0.79-3.37	0.19			
Concurrent chemotherapy	0.78	0.14-4.49	0.78			
Non-VMAT delivery	0.43	0.15-1.25	0.12			
Multiple RT courses	0.43	0.15-1.20	0.11			

LAD = left anterior descending coronary artery; LCx = left circumflex coronary artery; LMCA = left main coronary artery; MHD = mean heart dose; RCA = right coronary artery; RT = radiotherapy; TotalCor = all coronary arteries combined; other abbreviations as in Table 1.

**Figure 2:**
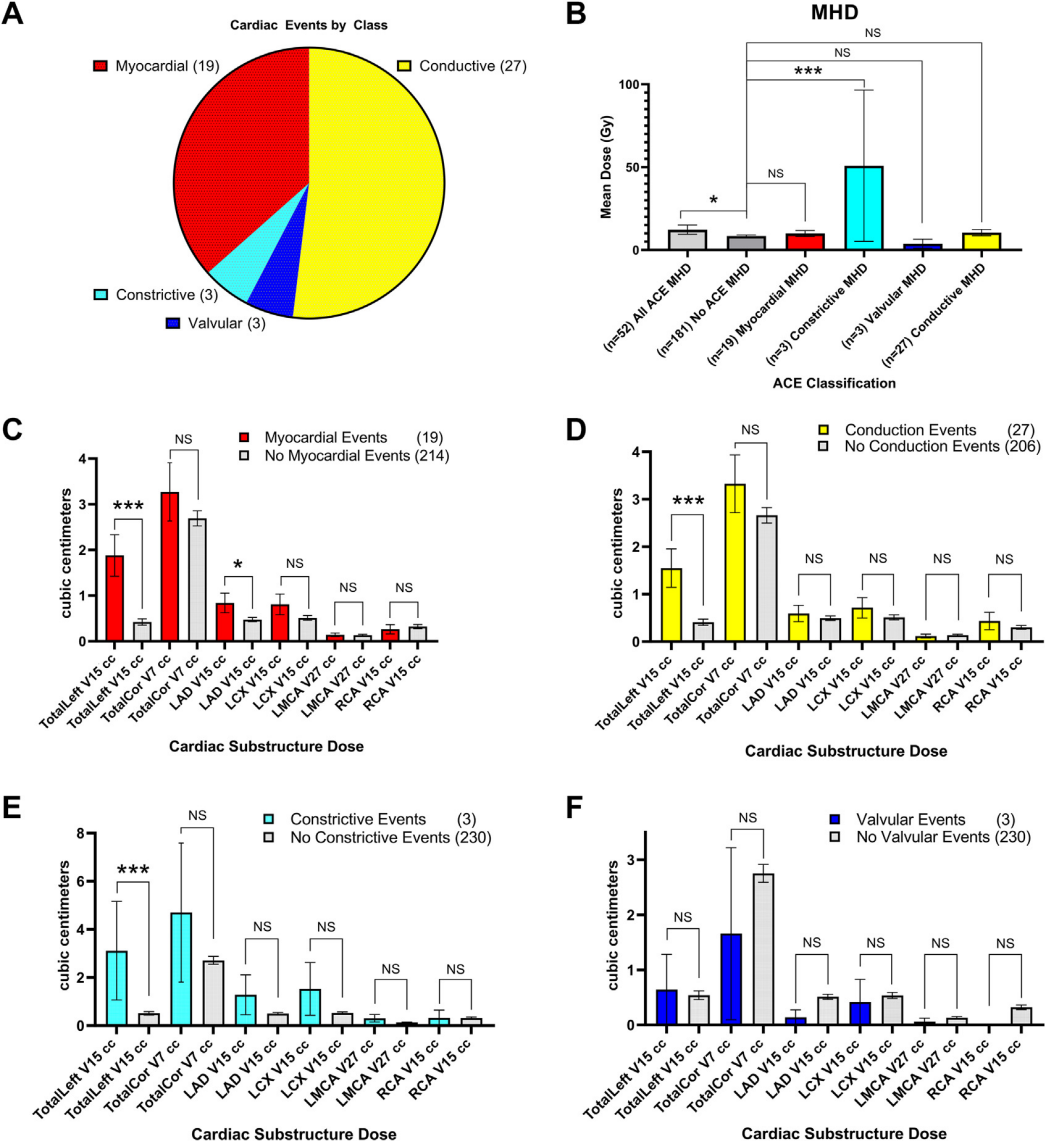
Cardiac Events and Associated Radiation Dose Factors (A) Among all patients, 22.3% experienced at least 1 cardiac event following thoracic radiotherapy; the majority of events were myocardial or conduction events. (B) Increased mean heart dose (MHD) was associated with an increased incidence of grade ≥3 adverse cardiac events (ACEs) (*P* = 0.040) and constrictive events (*P* < 0.010). (C to F) Increased left-sided coronary artery dose (TotalLeft) was significantly associated with myocardial (*P* < 0.01), conduction (*P* < 0.01), and constrictive (*P* < 0.010) cardiac events. With the exception of increased LAD doses association with myocardial events (*P* = 0.033), there were no other significant associations. ∗*P* < 0.05, ∗∗*P* < 0.01, and ∗∗∗*P* < 0.001. TotalCor = all coronary arteries combined; V7 = volume receiving 7 Gy; V15 = volume receiving 15 Gy; V27 = volume receiving 27 Gy; other abbreviations as in Figure 1.

**Figure 3:**
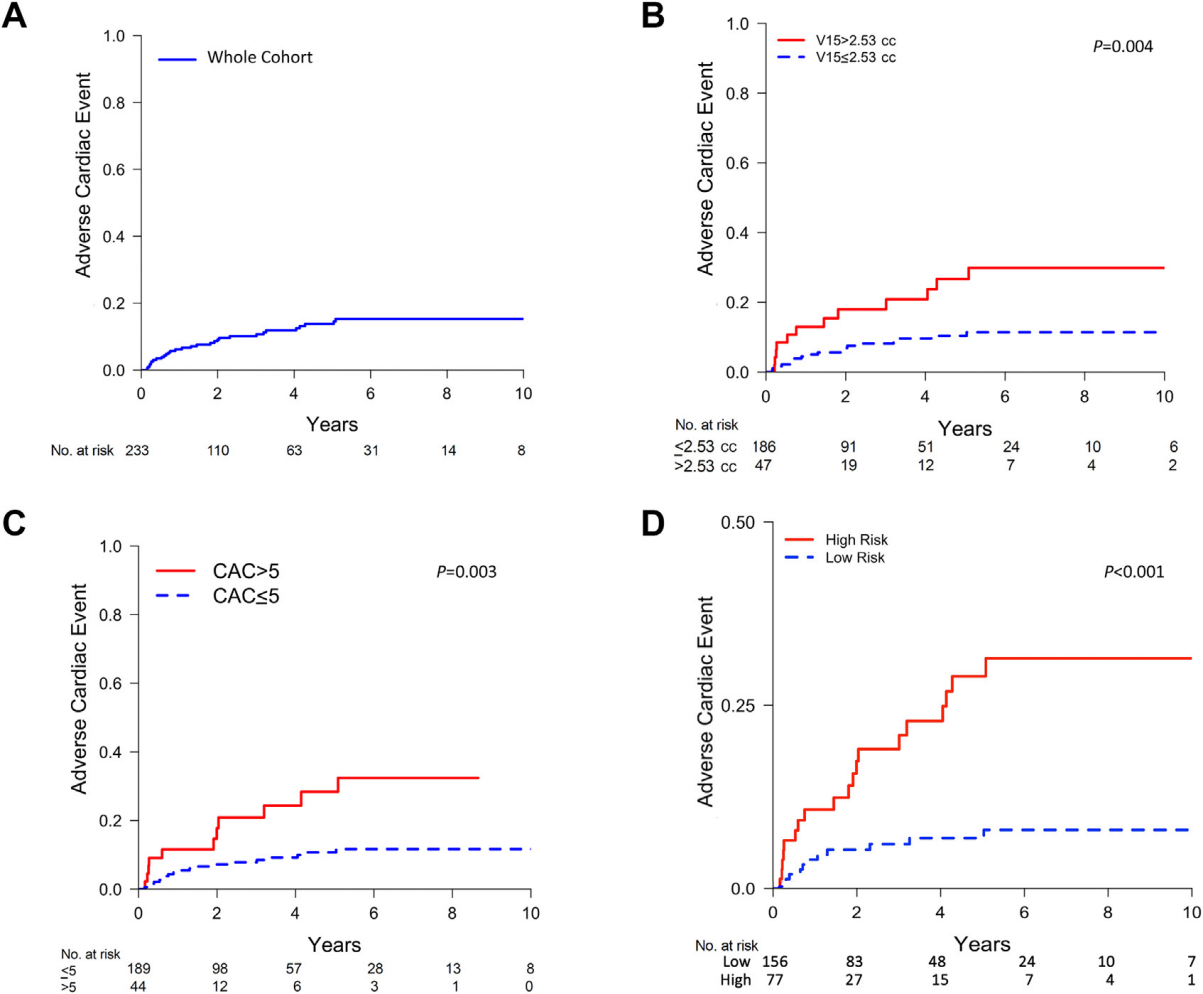
Cardiac Event Cumulative Incidence Curves Adjusted for Noncardiac Death (A) Cumulative incidence of adverse cardiac events with median time to the first cardiac event following radiotherapy of 21.5 months. The 5-year cardiac event incidence was 13.8% (95% CI: 10.6%-17.0%). (B) Cardiac event incidence for left coronary arteries (TotalLeft) receiving high (V15 >2.53 cc) vs low (V15 ≤2.53 cc) radiotherapy dose, with a 5-year incidence of 26.7% vs 10.6%, respectively. (C) Adverse cardiac event incidence in high (score >5) vs low (score ≤5) CAC score to TotalLeft, with a 5-year cardiac event incidence of 28.4% vs 10.7%, respectively. (D) Cumulative risk estimates when stratifying by linear function of fixed covariate values obtained with our competing-risk regression model including age, TotalLeft CAC score >5, and TotalLeft V15. The 5-year incidence of adverse cardiac events was 6.9% for the low-risk group and 28.9% for the high-risk group. Abbreviations as in Figure 2.

Nearly one-third of all patients (30.5% [n = 71]) had histories of cardiac events, and one-half of this subgroup (49.3% [n = 35]) had multiple cardiac events before initiating thoracic RT. Within the entire cohort, 22.3% (n = 52) experienced at least 1 cardiac event following RT: 19 were myocardial, 3 were constrictive, and 3 were valvular, and 27 patients experienced conduction events (Figure 2A). The median time to the first cardiac event post-RT was 21.5 months (range: 1.7-118.9 months). For the entire cohort, we observed a 5-year incidence of adverse cardiac events at 13.8% (95% CI: 13.0%-14.5%) (Figure 3A).

### CAC scoring

Regarding CAC scoring, among all the CT images, 14.6% (n = 34) included intravenous contrast, which limited the use of automated Agatston scoring. Thus, a modified visual scoring method^17^ was used for CAC scoring. Individual CAC scores are listed in Supplemental Table 1. In total, 52 (22.3%), 62 (26.6%), 58 (24.9%), and 61 (26.2%) patients had no, mild, moderate, and severe CAC, respectively (Table 1). On comparison testing, visual CAC scoring showed a significant correlation with automated Agatston scoring in a subcohort with noncontrast CT scans (*r* = 0.72; *P* < 0.001) (Supplemental Figure 2).

### Dose toxicity, CAC toxicity, and threshold modeling

Patients who experienced cardiac events received a significantly higher MHD compared with others (12.3 Gy vs 8.5 Gy; *P* = 0.040) (Figure 2B). Similarly, patients experiencing constrictive events received a significantly higher MHD compared with others (50.9 Gy vs 8.5 Gy; *P* < 0.01) (Figure 2C). Patients who experienced myocardial, conduction, and constrictive events had significantly greater left-sided coronary artery involvement of TotalLeft V15: 1.9 cc vs 0.4 cc (*P* < 0.01), 1.6 cc vs 0.4 cc (*P* < 0.01), and 3.1 cc vs 0.5 cc (*P* < 0.01), respectively, compared with those who did not experience such events. Only dose to the LAD was significantly associated with myocardial events (0.8 cc vs 0.5 cc; *P* = 0.03) (Figures 2C to 2F).

The obtained AUCs for the prediction performance of adverse cardiac events, on the basis of the mean dose to cardiac substructures, were 0.63, 0.59, 0.58, 0.58, 0.57, 0.57, 0.56, and 0.49 for the LV, LCx, TotalLeft, MHD, LAD, LMCA, TotalCor, and RCA, as determined by time-dependent ROC modeling at 5 years post-RT (Supplemental Table 2). Because the RCA dose, either alone or as part of TotalCor, did not improve performance, it was excluded for further thresholding analyses. For CAC score performance, we obtained AUC values of 0.64 for the LCx, 0.62 for TotalLeft, 0.60 for the LMCA, and 0.56 for the LAD.

Subsequently, we identified absolute dose constraints in cubic centimeters to the coronary arteries, because mean doses may be influenced by anatomical variation and user delineation. To identify rational thresholds and make comparisons with mean doses reported by others,^10^ we used the log-rank statistic to perform threshold modeling. The following optimal cutpoints were identified for mean doses: 15.5 Gy for the LAD, 13.2 Gy for the LMCA, 1.2 Gy for the LCx, and 1.27 Gy for TotalLeft. We chose to quantify V15 thresholds for the LMCA, LCx, LAD, TotalLeft, and LV. Subsequently, the chosen thresholds were based on previously reported mean threshold values,^10^ our mean threshold values with potential alternative cutpoints (Supplemental Figure 3), and the choice of 15 Gy as it represents a readily implementable value in clinical settings. We found the following V15 thresholds: 1.53 cc for the LAD, 0.16 cc for the LMCA, 0.71 cc for the LCx, 2.53 cc for TotalLeft, and 36.28 cc for the LV. We identified a CAC cutpoint score of >5 for TotalLeft. Furthermore, when excluding patients who underwent multiple courses of RT, we obtained nearly identical V15 thresholds: 1.53 cc for the LAD, 0.15 cc for the LMCA, 1.59 cc for the LCx, 3.52 cc for TotalLeft, and 5.48 cc for the LV. However, there was a local maximum threshold at 36 cc (Supplemental Figure 4).

We then evaluated whether including CAC and dose metrics could improve model performance. Given the minimal difference in AUC values for univariable models predicting cardiac events including either MHD or coronary artery substructures, we also wanted to determine whether a multivariable model including CAC might further provide evidence for improved prediction when including coronary artery dose information compared with MHD alone. We repeated our time-dependent ROC analyses at the 5-year mark post-RT for the left coronary arteries’ V15 (milliliters), and the results are shown in Supplemental Table 2.

The composite model combining TotalLeft CAC and V15 (milliliters) showed the highest performance, with an AUC of 0.69 (95% CI: 0.59-0.80), compared with the AUCs for individual coronary arteries (Supplemental Table 2, Supplemental Figure 5). This composite model was higher than both MHD and TotalLeft CAC (AUC: 0.64; 95% CI: 0.54-0.75).

### Competing-risk regression analysis

We performed both univariable and multivariable competing-risk regression analyses, adjusting for the competing risk for noncardiac death. In the univariable analysis, age, available in all patients, was the only baseline clinical factor significantly associated with cardiac events (Table 2). Although only a few of the patients had calculated ASCVD scores, this showed an association with adverse cardiac events (HR: 1.14; 95% CI: 1.03-1.25; *P* = 0.008). Nearly all RT dose and CAC factors showed significant associations with cardiac events (Table 2); however, mean RCA dose was not significantly associated with cardiotoxicity, similar to findings from Atkins et al.^10^ Given these results together with a low AUC value, we did not include RCA dose or CAC for subsequent analyses. On the basis of our ROC and thresholding analyses, we included the volume of 15 Gy received by the LMCA, LAD, and LCx combined (TotalLeft V15) per 1 cc as the dose variable for multivariable analyses. In multivariate analysis, after adjusting for age, we observed that increasing TotalLeft V15 (per 1 cc; HR: 1.38; 95% CI: 1.11-1.72; *P* = 0.004) as well as a calcium score >5 of the LMCA, LAD, and LCx combined (TotalLeft CAC score > 5; HR: 2.51; 95% CI: 1.08-5.86; *P* = 0.033) were both significantly associated with cardiac events (Table 2). There was no significant interaction between TotalLeft CAC score >5 and TotalLeft V15 (*P* = 0.23).

### Cumulative incidence of grade ≥ 3 cardiac events by dose and CAC

We observed a higher incidence of adverse cardiac events over a 5-year period (26.7%; 95% CI: 22.1%-31.5%) in patients who received higher doses to the left-sided coronary vessels (LAD, LMCA, and LCx), in contrast to the incidence (10.6%; 95% CI: 9.6%-11.2%) observed in patients with lower doses to these vessels (TotalLeft V15 ≤2.53 cc) in their respective dose groups (Figure 3B) (*P* = 0.004). Furthermore, we observed a higher incidence of cardiac events in patients with high CAC scores (>5) attaining 28.4% (95% CI: 23.0%-34.0%), compared with an incidence of 10.7% (95% CI: 9.9%-11.5%) in those with low CAC scores (≤5) (Figure 3C) (*P* = 0.003). A TotalLeft CAC score >5 represents either moderate CAC in all 3 vessels, heavy CAC in 2, or heavy CAC in 1 vessel paired with moderate and mild CAC in the other 2 vessels. To develop a risk score model incorporating TotalLeft CAC and V15, we used multivariable regression model coefficients from Table 2, including age (continuous), TotalLeft V15 (per milliliter), and TotalLeft CAC score >5 (binary), to calculate the value of the linear function of fixed covariates using our competing-risk regression model. Subsequently, a log-rank threshold for the competing-risk regression model values defined a low-risk group (regression model, raw value ≤13.15) and a high-risk group (raw value >13.15). A higher 5-year incidence of events was observed for the high-risk group (28.9%; 95% CI: 26.0%-32.1%) compared with the low-risk group (6.9%; 95% CI: 6.2%-7.7%), as shown in Figure 3D (*P* < 0.001).

Additionally, there was no significant difference in cardiac events in those with prior RT vs without prior RT that exceeded TotalLeft dose constraints (*P* = 0.49) or in cardiac events when TotalLeft dose constraints were not exceeded (*P* = 0.052) (Supplemental Figure 6).

## Discussion

We investigated the risk factors for grade ≥3 adverse cardiac events after RT for locally advanced NSCLC, observing strong associations between coronary artery dose and pretreatment CAC score with the occurrence of cardiac events. With a median follow-up period of more than 6 years, we observed a 5-year cardiac event incidence of 13.8% following thoracic RT (Central Illustration). These cardiac events spanned a time frame of 1.7 to 118.9 months, with an IQR of 32.4 months (IQR: 7.1-39.5 months). Although the occurrence of such subacute events might not be expected soon after RT, we opted to keep these early events in our analysis to inform the characterization of potential etiologies of cardiotoxicity. Nearly all of the observed cardiac events (88.5%) were either conduction or myocardial. Although high doses to the sinoatrial node have been associated with atrial fibrillation development,^11^ preclinical evidence demonstrates that ischemia and infarctions represent major inciting events for remodeling mechanisms, such as fibrosis, for the development of arrhythmias.^24^^,^^25^ Moreover, ischemia has been associated with the onset of arrhythmias after RT.^26^ Therefore, to identify subgroups in our cohort at high risk for cardiotoxicity, we used absolute dose volume metrics to obtain dose constraints for individual coronary arteries. This approach revealed an approximate 2.5-fold increase in cardiac event incidence associated with elevated doses in the left coronary artery (V15 >2.53 cc). Additionally, we identified that CAC imaging biomarkers were associated with cardiac events in a dose-dependent manner. In a composite model, we identified low- and high-risk subgroups by including factors such as increasing age, TotalLeft CAC, and V15. This improved understanding of vessel dosimetry and baseline vessel health provides valuable insights to reduce long-term cardiac damage for individuals undergoing RT.

**Central Illustration:**
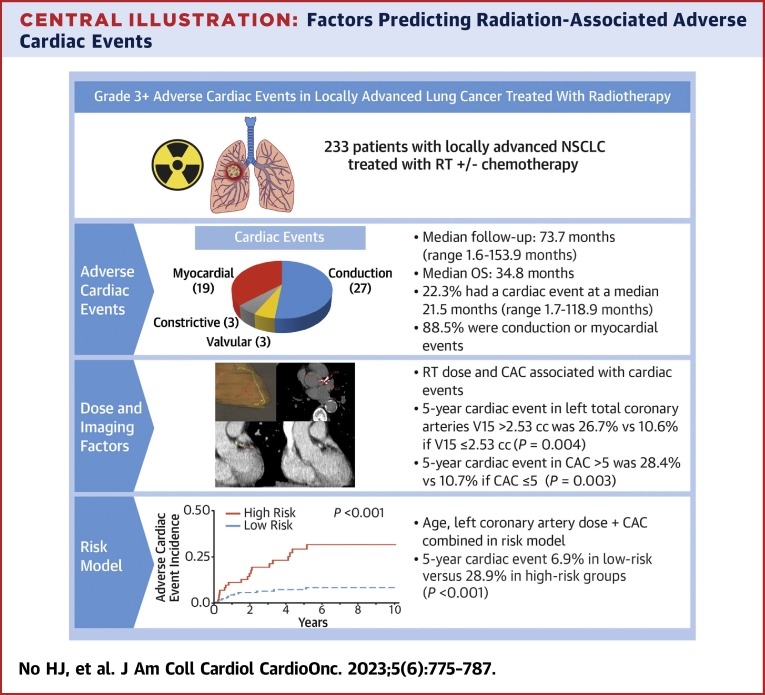
Factors Predicting Radiation-Associated Adverse Cardiac Events In a median follow-up of 73.7 months, radiotherapy (RT)–associated grade ≥3 adverse cardiac events occurred in 22.3% of those receiving thoracic RT in a median time of 21.5 months. A 5-year cardiac event incidence of 26.7% vs 10.6% (*P* = 0.004) was seen for high- vs low-dose groups, where left-sided coronary artery volume receiving 15 Gy (V15) exceeding 2.53 cc was considered high. Additionally, high coronary artery calcium (CAC) (score >5) exhibited 28.4% vs 10.7% cardiac events compared with a low score. An integrated model incorporating dose and CAC showed a higher 5-year adverse cardiac event incidences of 6.9% and 28.9% for low-risk vs high-risk patients, respectively. NSCLC = non–small cell lung cancer; OS = overall survival.

For decades, elevated MHD was used as a dose constraint during RT planning.^4^ Illustrating the linear increase in cardiotoxicity risk with higher MHD, Darby et al^4^ reported a 7.4% increase in cardiac events per mean dose delivered to the heart. However, recent studies have demonstrated that cardiac substructure doses have improved predictive performance for cardiotoxicity.^10^^,^^11^^,^^27^ Although we did find an association between MHD and cardiac events in our study, our analysis showed that dose to the left coronary arteries and CAC score had the strongest predictive performance on the basis of ROC analyses. Furthermore, dose to the LV also displayed predictive capability for cardiac events; however, when combined with CAC, it performed less well compared with dose to the left coronary arteries. The consistency of our findings with the work by Atkins et al^10^ is noteworthy. They showed in a cohort of 701 patients that RT doses received by the left coronary arteries had a higher AUC for cardiac events than MHD and were significantly associated with cardiac events. We externally validated the mean dose constraints reported by Atkins et al^10^ and obtained a nearly identical mean dose threshold for the LAD (∼15 Gy). Given their large cohort size and overlapping potential dose thresholds seen in our cohort (Results and Supplemental Figure 3), we identified dose constraints for the volume in milliliters of left coronary arteries receiving doses >15 Gy. We opted to provide absolute volumetric dose constraints because user-defined structures and anatomical variation may result in less reliable mean or percentage doses.

Of note, we aimed to provide a comprehensive overview of grade ≥3 adverse cardiac events, which may not be limited to previous definitions, as highlighted by Atkins et al.^10^ For example, in the cohort reported by Atkins et al,^10^ only approximately 10% of patients experienced cardiac events, but it is unknown how many subsequently developed other cardiac complications that may be secondary to RT exposure of critical structures. In fact, our own data exhibited a similar 8.2% rate of events when considering the same event categories. However, a substantial number of other cardiac events, such as the development of atrial fibrillation, suggests that there is a higher occurrence of these clinically meaningful events that also appear to be related to dose received by cardiac structures.

Although doses to the left coronary arteries emerge as factors significantly associated with cardiotoxicity and that may be optimized during radiation treatment planning, we also studied baseline patient risk factors. This included evaluating CAC scores, which could serve as imaging biomarkers for pre-RT cardiotoxicity risk assessment, thus improving risk stratification.^12^^,^^13^ Although Kim et al^11^ did not observe a significant association between the presence of calcification and atrial fibrillation, our results suggest that a CAC severity score provides a strong association with cardiac events. Notably, our cohort recorded a large number of arrhythmia events (see Figures 3C and 3D and Table 2 with optimized cutpoint for CAC). The association between cardiotoxicity with CAC has been further supported in studies by Wang et al^12^ and Gal et al.^13^ In a multivariable ROC model that included both left coronary artery CAC score and TotalLeft V15, we observed an AUC of 0.69, suggesting that these 2 independent risk factors in combination may deserve further study.

Given the strong association between CAC and cardiotoxicity, it is necessary to establish standardized approaches to compute CAC score. Although computationally intensive voxel-calculated scoring approaches have been used,^13^^,^^17^^,^^21^ visual CAC scoring methods, such as the method of Chiles et al^17^ we validated, have generated comparable risk assessments. We observed a high correlation between the visual scoring method and Agatston scoring (Supplemental Figure 2). Thus, our visual scoring assessment represents a clinically implementable tool that may be used for risk stratification, further justifying RT dose reduction for left coronary arteries or early medical risk reduction strategies when dose reduction is not possible.^12^^,^^28^, ^29^, ^30^ Improved image guidance and techniques such as respiratory and cardiac gating could aid in treatment planning to reduce doses to regions with severe calcifications and could further help mitigate cardiotoxicity, as seen in breast cancer RT, in which doses to calcified regions were associated with increased cardiotoxicity.^31^ However, because of the 3-dimensional nature of lung RT involving all 3 left coronary arteries, implementing optimal dosing to severe calcifications would likely present challenges.

Our study provides not only valuable external validation of the strong association between the RT dose received by the left coronary arteries and adverse cardiac events^10^ but also new evidence supporting the utility of CAC scoring in the stratification of patient risk prior to treatment. Our data may be leveraged in the design of future prospective clinical trials. In fact, there is growing recognition of the necessity for prospective data to incorporate cardiotoxicity endpoints into trial design.^32^ The landmark study by Darby et al,^4^ which highlighted the association of cardiac dose and cardiotoxicity, was followed by a study of more than 1,800 trials that showed no difference in the inclusion of cardiotoxicity endpoints between the pre-Darby and post-Darby era (3.9% vs 5.9%; *P* = 0.46),^32^ highlighting the lack of prospective data and shedding light on future directions to inform personalized RT practices.

### Study limitations

Our study has several limitations, including its retrospective nature, limited sample size, and lack of external validation cohorts. Although we do not have validation cohorts for our CAC scoring method, our dose-volume data serve as some validation of the findings of Atkins et al,^10^ suggesting the robustness of our derived dose constraints. Of note, our ROC analyses exhibited a relatively overall modest AUC of 0.69 for the TotalLeft, which could likely be improved upon with prospective clinical trials designed to more robustly capture cardiac events and baseline cardiac risk characteristics. However, these values were similar to that of Atkins et al,^10^ who reported an AUC of 0.59 for the total coronary artery structure.

Furthermore, because the dependence on user-defined coronary artery contouring introduces a potential source of variability, we attempted to mitigate this using absolute volume dose thresholds. Additionally, the CT planning image sets did not use cardiac gating, and some included intravenous contrast, leading to image quality variability. Finally, CAC scoring was performed through visual assessment, which may be subject to inherent biases. However, our comparison with automated computerized scoring did show comparability.

Our cohort included 57 (24.5%) patients who had undergone multiple courses of thoracic RT; however, all grade ≥3 cardiac events occurred after the delivery of all RT courses. When excluding these patients from our dose constraint threshold modeling, we obtained nearly identical dose thresholds for the coronary arteries compared with the overall cohort. Additionally, through competing-risk regression (Table 2), we did not observe a higher risk for cardiotoxicity in individuals with prior RT. Finally, the incidence of cardiac events among those with prior RT compared with those without, exceeding TotalLeft dose constraints, did not show a significant difference (Supplemental Figure 6). This suggests that including these patients in our analysis does not appear to introduce bias to our results; in fact, their inclusion may provide valuable additional data on dose constraints when accumulating doses across multiple courses of RT.

Finally, we were surprised to find no association between traditional CV risk factors and prior CV disease and outcomes. However, this was likely due to the limited sample size of our cohort and its homogenous nature. The prevalence of smokers and a large number of other high-risk factors among those evaluated may limit the correlation of traditional CV risk factors.

## Conclusions

Radiation-associated grade ≥3 adverse cardiac events occur in more than 20% of individuals undergoing thoracic RT within a median time frame of <2 years. We observed an elevated cardiac event risk following thoracic RT, particularly for patients with moderate to severe CAC who receive elevated RT doses to the left coronary arteries (TotalLeft V15 >2.53 cc). Our RT dose constraints offer external validation to findings in other large cohorts, and our CAC scoring provides new clinical evidence for assessing the baseline risk for cardiotoxicity prior to treatment. With further study, our findings may serve to inform personalized RT planning and facilitating early medical intervention for patients with locally advanced NSCLC who are undergoing thoracic RT.

Perspectives **COMPETENCY IN MEDICAL KNOWLEDGE:** In patients with lung cancer treated with thoracic RT, measured pretreatment imaging of CAC alongside RT dose to specific cardiac substructures are associated with adverse cardiac events, which occurred in >20% of our cohort at a median time of <2 years.

**TRANSLATIONAL OUTLOOK:** Future studies are needed to assess the utility of measuring these markers of cardiac risk in those undergoing definitive thoracic RT to inform personalized treatment strategies.

## Funding Support and Author Disclosures

This work was supported by a Radiological Society of North America Research & Education Foundation Resident Research Grant. Dr No has received research grant funding from the Radiological Society of North America. Dr Kastelowitz has received research grant funding from the American Society for Radiation Oncology–LUNGevity and the Radiological Society of North America; and has received consulting fees from MIM Software. Dr Rhee has received funding from the National Institutes of Health (grant K08-HL148540); and has conducted industry-sponsored research for Pfizer. Dr Clark has received research grant funding from the Adult Congenital Heart Association. Dr Vitzthum has received research grants from RefleXion. Dr Loo has received research grants from the National Institutes of Health (grant P01CA244091) and Varian Medical Systems; holds stock in TibaRay; and is a board member for TibaRay. Dr Diehn has received research grant funding from Varian, Genentech, and AstraZeneca; receives royalties for patent licenses from Roche and Foresight Diagnostics; has received consulting fees from Roche, Varian, BioNTech, RefleXion, Novartis, Illumina, Genentech, Boehringer Ingelheim, and Gritstone Oncology; licenses patents to Roche and Foresight Diagnostics for liquid biopsy methods and to Celgene for single-cell analysis methods; is on advisory boards for AstraZeneca, Genentech, Boehringer Ingelheim, Illumina, and Gritstone Oncology; is a board member for Foresight Diagnostics; holds stock in Foresight Diagnostics and CiberMed; and receives in-kind research reagents from Illumina. All other authors have reported that they have no relationships relevant to the contents of this paper to disclose.

## References

[1] National Cancer Institute Common Terminology Criteria for Adverse Events (CTCAE): protocol development: CTEP. https://ctep.cancer.gov/protocoldevelopment/electronic_applications/ctc.htm.

[2] Mulrooney D.A., Yeazel M.W., Kawashima T. (2009). Cardiac outcomes in a cohort of adult survivors of childhood and adolescent cancer: retrospective analysis of the Childhood Cancer Survivor Study cohort. BMJ.

[3] Dess R.T., Sun Y., Matuszak M.M. (2017). Cardiac events after radiation therapy: combined analysis of prospective multicenter trials for locally advanced non-small-cell lung cancer. J Clin Oncol.

[4] Darby S.C., Ewertz M., McGale P. (2013). Risk of ischemic heart disease in women after radiotherapy for breast cancer. N Engl J Med.

[5] Taylor C., Correa C., Duane F.K. (2017). Estimating the risks of breast cancer radiotherapy: evidence from modern radiation doses to the lungs and heart and from previous randomized trials. J Clin Oncol.

[6] Niska J.R., Hu J., Li J. (2021). Using novel statistical techniques to accurately determine the predictive dose range in a study of overall survival after definitive radiotherapy for stage III non-small cell lung cancer in association with heart dose. J Cancer Ther.

[7] van Nimwegen F.A., Schaapveld M., Cutter D.J. (2016). Radiation dose-response relationship for risk of coronary heart disease in survivors of Hodgkin lymphoma. J Clin Oncol.

[8] van den Bogaard V.A.B., Ta B.D.P., van der Schaaf A. (2017). Validation and modification of a prediction model for acute cardiac events in patients with breast cancer treated with radiotherapy based on three-dimensional dose distributions to cardiac substructures. J Clin Oncol.

[9] Hoppe B.S., Bates J.E., Mendenhall N.P. (2020). The meaningless meaning of mean heart dose in mediastinal lymphoma in the modern radiation therapy era. Pract Radiat Oncol.

[10] Atkins K.M., Chaunzwa T.L., Lamba N. (2021). Association of left anterior descending coronary artery radiation dose with major adverse cardiac events and mortality in patients with non-small cell lung cancer. JAMA Oncol.

[11] Kim K.H., Oh J., Yang G. (2022). Association of sinoatrial node radiation dose with atrial fibrillation and mortality in patients with lung cancer. JAMA Oncol.

[12] Wang K., Malkin H.E., Patchett N.D. (2022). Coronary artery calcifications and cardiac risk after radiation therapy for stage III lung cancer. Int J Radiat Oncol Biol Phys.

[13] Gal R., van Velzen S.G.M., Hooning M.J. (2021). Identification of risk of cardiovascular disease by automatic quantification of coronary artery calcifications on radiotherapy planning CT scans in patients with breast cancer. JAMA Oncol.

[14] Greenland P., Blaha M.J., Budoff M.J., Erbel R., Watson K.E. (2018). Coronary calcium score and cardiovascular risk. J Am Coll Cardiol.

[15] Shemesh J., Henschke C.I., Shaham D. (2010). Ordinal scoring of coronary artery calcifications on low-dose CT scans of the chest is predictive of death from cardiovascular disease. Radiology.

[16] Phillips W.J., Johnson C., Law A. (2019). Comparison of Framingham risk score and chest-CT identified coronary artery calcification in breast cancer patients to predict cardiovascular events. Int J Cardiol.

[17] Chiles C., Duan F., Gladish G.W. (2015). Association of coronary artery calcification and mortality in the national lung screening trial: a comparison of three scoring methods. Radiology.

[18] Detterbeck F.C., Boffa D.J., Kim A.W., Tanoue L.T. (2017). The eighth edition lung cancer stage classification. Chest.

[19] Lloyd-Jones D.M., Braun L.T., Ndumele C.E. (2019). Use of risk assessment tools to guide decision-making in the primary prevention of atherosclerotic cardiovascular disease: a special report from the American Heart Association and American College of Cardiology. J Am Coll Cardiol.

[20] Duane F., Aznar M.C., Bartlett F. (2017). A cardiac contouring atlas for radiotherapy. Radiother Oncol.

[21] Atkins K.M., Weiss J., Zeleznik R. (2022). Elevated coronary artery calcium quantified by a validated deep learning model from lung cancer radiotherapy planning scans predicts mortality. JCO Clin Cancer Inform.

[22] Solbak M.S., Henning M.K., England A., Martinsen A.C., Aaløkken T.M., Johansen S. (2020). Impact of iodine concentration and scan parameters on image quality, contrast enhancement and radiation dose in thoracic CT. Eur Radiol Exp.

[23] Bae K.T. (2010). Optimization of contrast enhancement in thoracic MDCT. Radiol Clin North Am.

[24] Tice B.M., Rodríguez B., Eason J., Trayanova N. (2007). Mechanistic investigation into the arrhythmogenic role of transmural heterogeneities in regional ischaemia phase 1A. Europace.

[25] Rodríguez B., Tice B.M., Eason J.C., Aguel F., Ferrero J.M., Trayanova N. (2004). Effect of acute global ischemia on the upper limit of vulnerability: a simulation study. Am J Physiol Heart Circ Physiol.

[26] Nabiałek-Trojanowska I., Lewicka E., Wrona A. (2020). Cardiovascular complications after radiotherapy. Cardiol J.

[27] Bergom C., Bradley J.A., Ng A.K. (2021). Past, present, and future of radiation-induced cardiotoxicity: refinements in targeting, surveillance, and risk stratification. J Am Coll Cardiol CardioOnc.

[28] Patel J., Pallazola V.A., Dudum R. (2021). Assessment of coronary artery calcium scoring to guide statin therapy allocation according to risk-enhancing factors: the Multi-Ethnic Study of Atherosclerosis. JAMA Cardiol.

[29] Mitchell J.D., Fergestrom N., Gage B.F. (2018). Impact of statins on cardiovascular outcomes following coronary artery calcium scoring. J Am Coll Cardiol.

[30] Tsiara S., Elisaf M., Mikhailidis D.P. (2003). Early vascular benefits of statin therapy. Curr Med Res Opin.

[31] van den Bogaard V.A.B., Spoor D.S., van der Schaaf A. (2021). The importance of radiation dose to the atherosclerotic plaque in the left anterior descending coronary artery for radiation-induced cardiac toxicity of breast cancer patients?. Int J Radiat Oncol Biol Phys.

[32] Prasad R.N., Miller E.D., Addison D., Bazan J.G. (2022). Lack of cardiotoxicity endpoints in prospective trials involving chest radiation therapy: a review of registered, latter-phase studies. Front Oncol.

