# Supplementary material for: Predicting Adverse Cardiac Events After Radiotherapy for Locally Advanced Non–Small Cell Lung Cancer
Source: JACC CardioOncol. 2023 Oct 4;5(6):775–87. doi: 10.1016/j.jaccao.2023.08.007 (PMC10774791; doi:10.1016/j.jaccao.2023.08.007)
Supplement: Supplemental Figures 1-6 and Supplemental Tables 1-3 [file mmc1.docx]

**Supplemental Appendix**

**Predicting Adverse Cardiac Events after Radiotherapy for Locally Advanced Non-Small Cell Lung Cancer**

**Content**

*Supplemental Figure 1. Clinical outcomes for the entire cohort of patients with locally advanced NSCLC* **Page 2**

*Supplemental Figure 2. Comparison of Visual CAC Scoring to Agatston Scoring* **Page 3**

*Supplemental Figure 3. Mean threshold values with potential alternate cutpoints* **Page 4**

*Supplemental Figure 4. Optimal cutpoint value for Left Ventricle V15Gy (cc)* **Page 5**

*Supplemental Figure 5. Composite model of TotalLeft CAC and V15 (cc)* **Page 6**

*Supplemental Figure 6. Grade ≥3 cardiac event incidence in patients with or without prior thoracic radiotherapy (RT) courses by TotalLeft dose.* **Page 7**

*Supplemental Table 1. Individual Coronary Artery Calcium Scores by Visual Scoring* **Pages 8-13**

*Supplemental Table 2. ROC analyses* **Page 14**

*Supplemental Table 3. Individual Systemic Therapy Courses* **Pages 15-20**


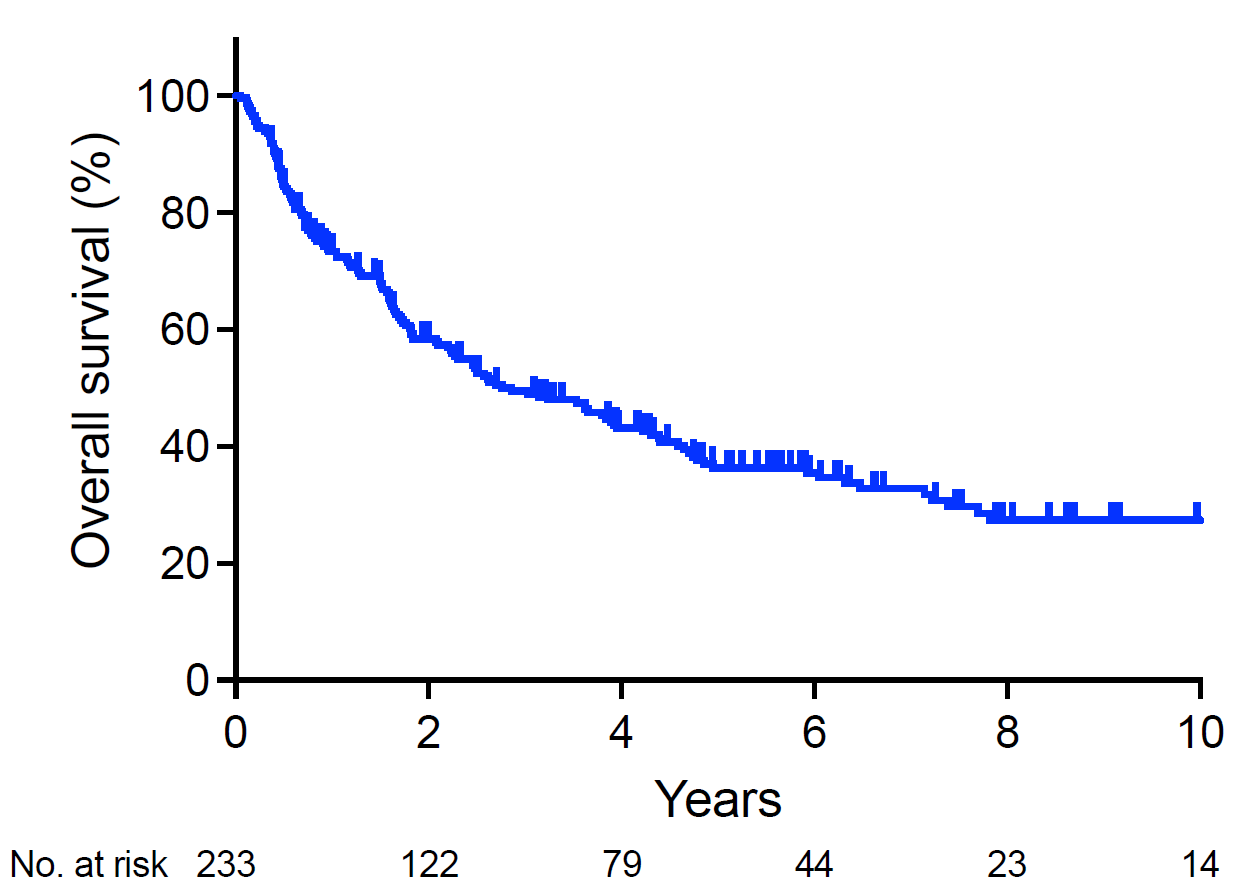


Supplemental Figure 1. Clinical outcomes for the entire cohort of patients with locally advanced NSCLC.

Kaplan Meier survival analysis with median overall survival of 34.8 months.


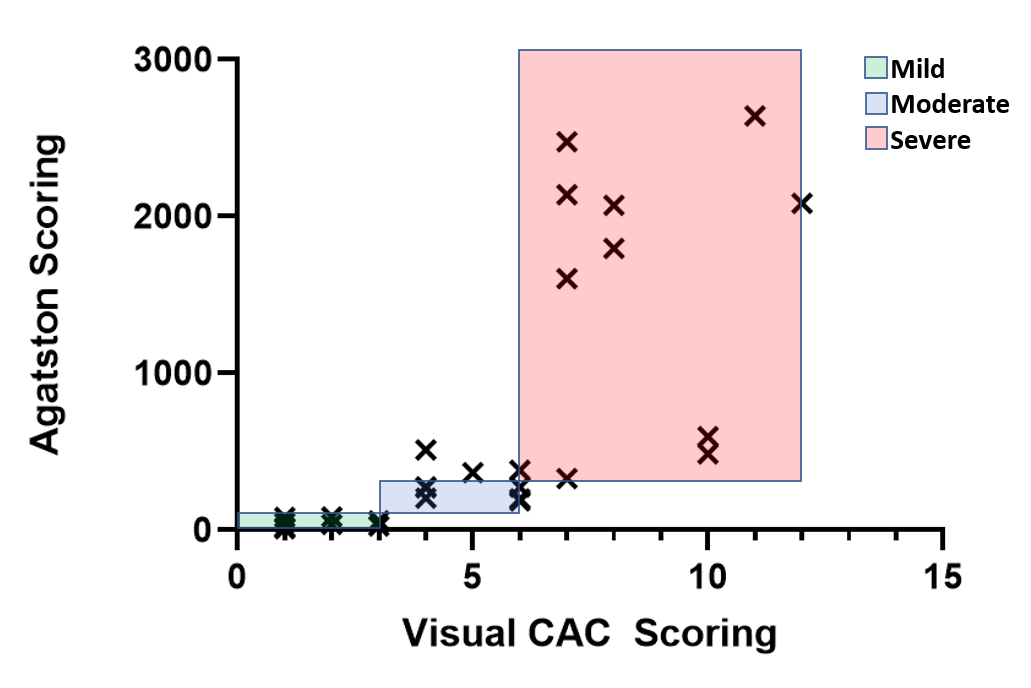


Supplemental Figure 2. Comparison of Visual CAC Scoring to Agatston Scoring

Visual CAC scoring was compared with established automated Agatston scoring using AVIEW CAC (Coreline Soft, Seoul, South Korea). Thirty visually scored mild, moderate, and severe cases, equally distributed, with non-contrast imaging, had Agatston scoring performed and compared. Visual CAC scoring showed significant correlation with automated Agatston scoring by Pearson correlation (r=0.72, *P<0.001*).


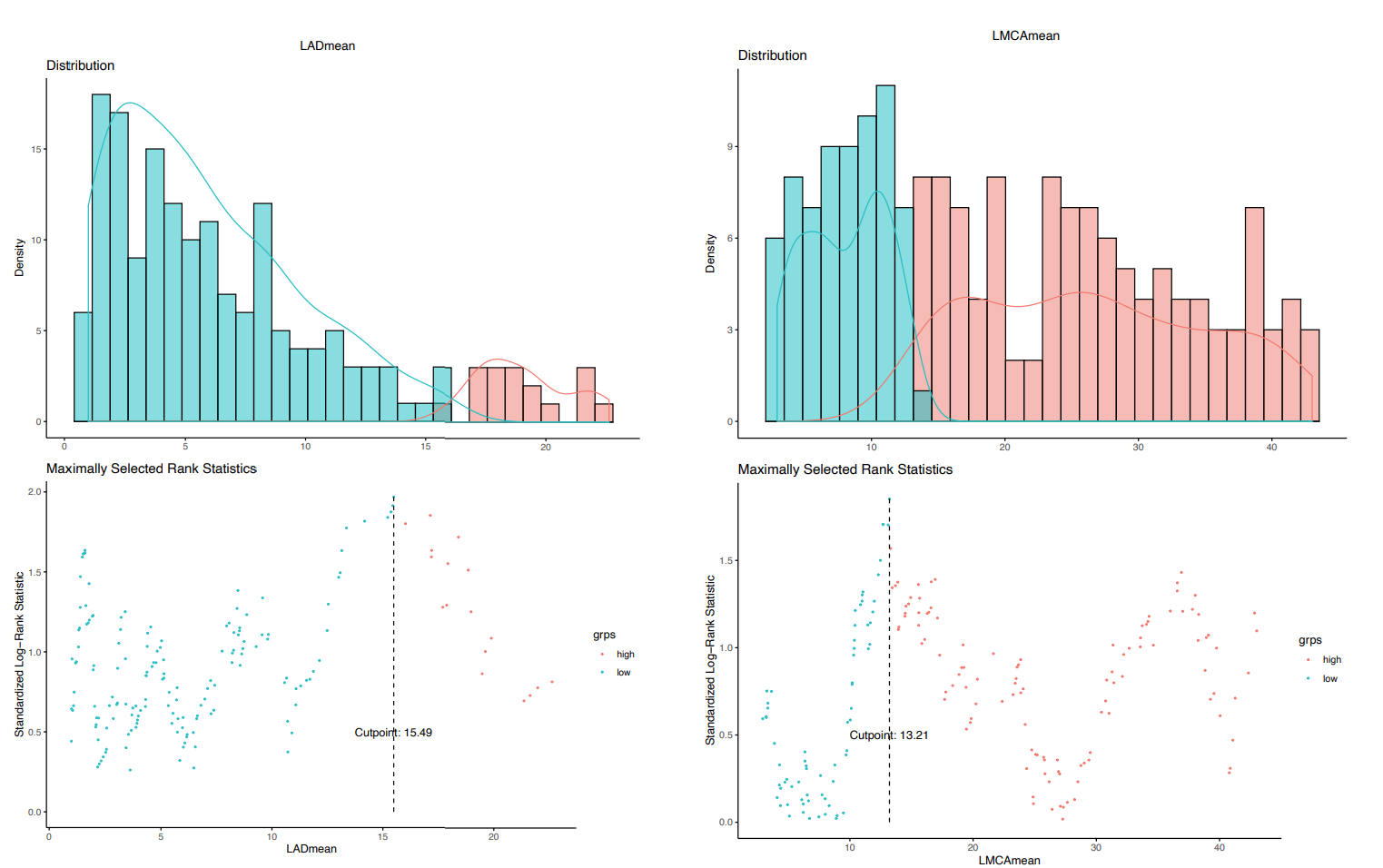


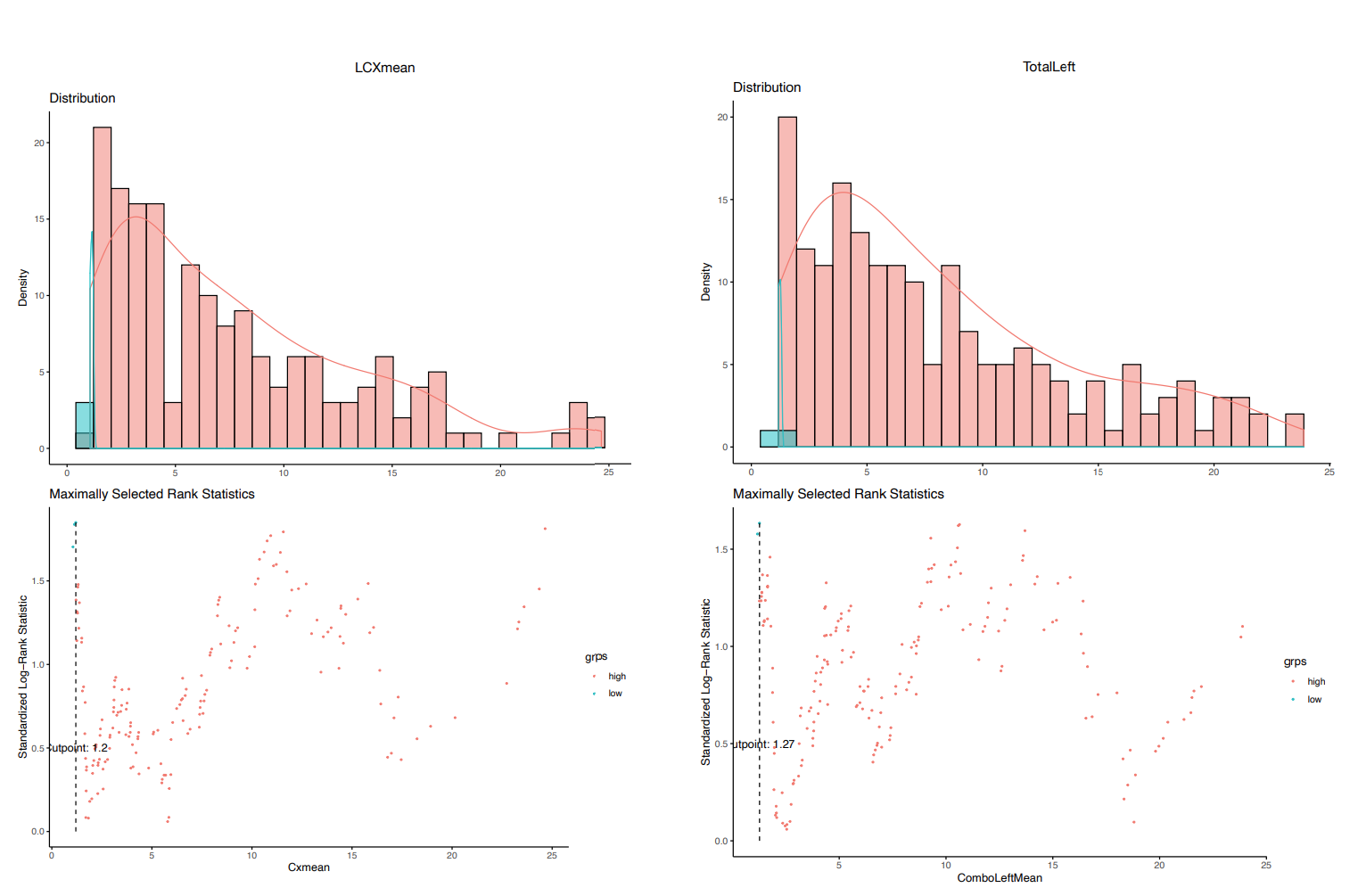


Supplemental Figure 3. Mean threshold values with potential alternate cutpoints.

Log-rank statistic values shown as histogram plots (top panels) and scatter plots (bottom panels) for various mean threshold values with potential cutpoints for the lower anterior descending (LAD) artery, left main coronary artery (LMCA), left circumflex (LCX), and TotalLeft (LAD+LMCA+LCX).


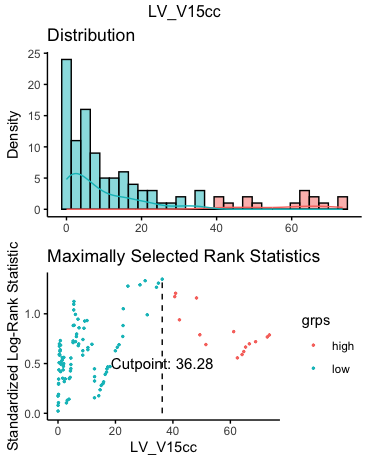


Supplemental Figure 4. Optimal cutpoint value for Left Ventricle V15Gy (cc)

Log-rank statistic values shown as histogram plots (top panel) and scatter plots (bottom panel) for the optimal cutpoint for the volume in cubic centimeters of the left ventricle (LV) receiving 15 Gray or higher (V15cc).


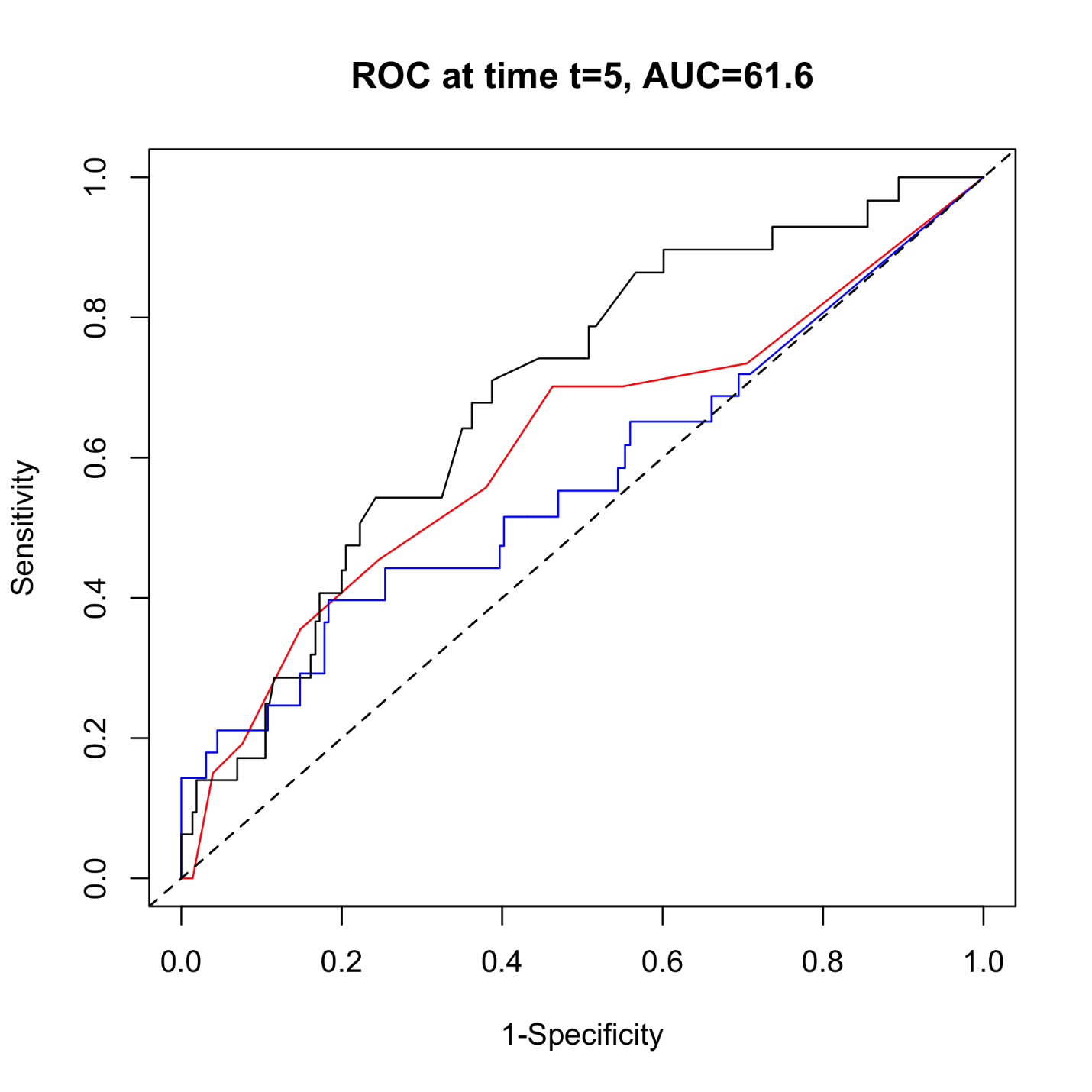


Supplemental Figure 5. Composite model of TotalLeft CAC and V15 (cc)

ROC curve at 5 years of a composite model of TotalLeft coronary artery calcium and V15 (cc) which had the highest performance with an AUC=0.69.


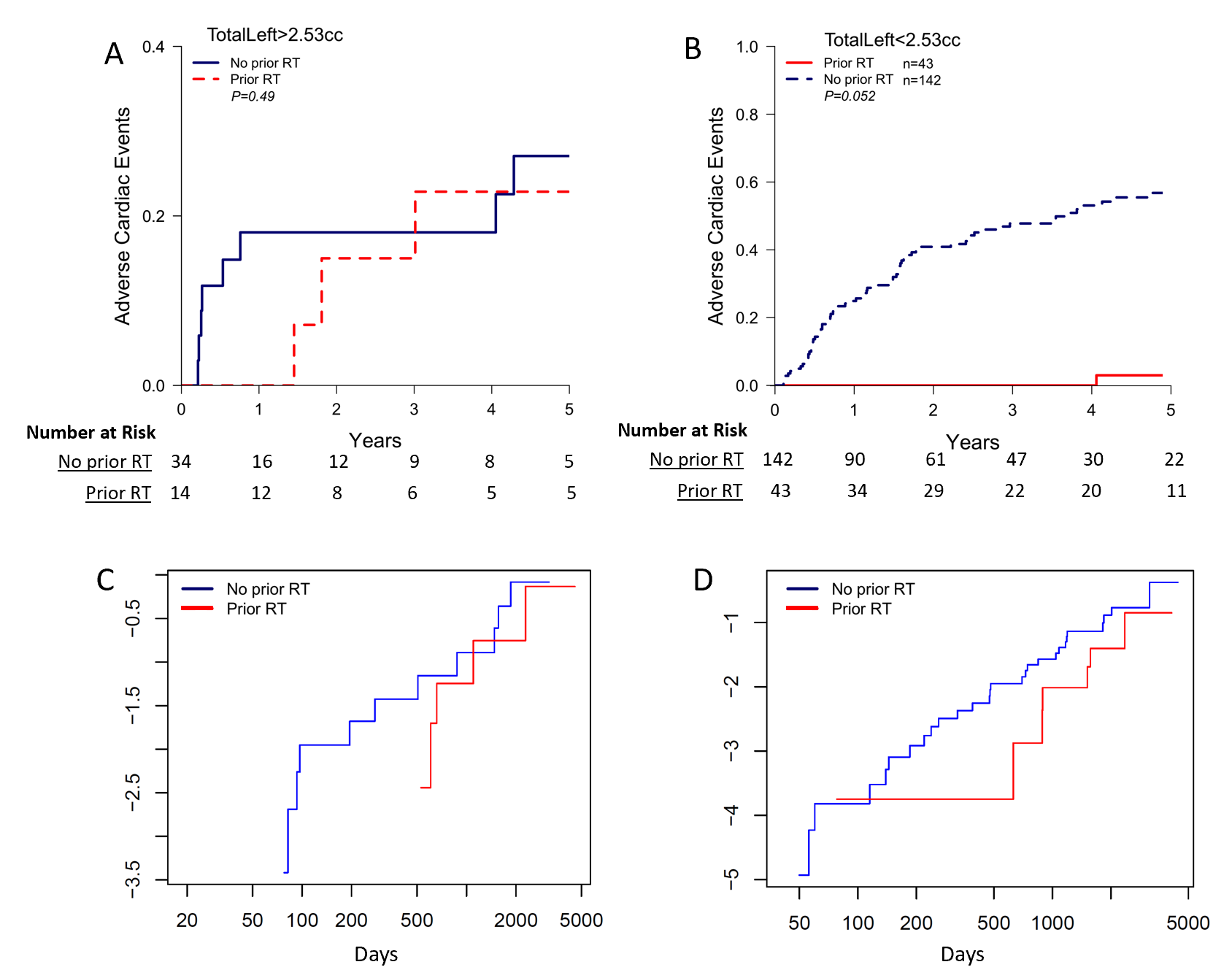


Supplemental Figure 6. Grade ≥3 cardiac event incidence in patients with or without prior thoracic radiotherapy (RT) courses by TotalLeft dose.

There were no significant differences in Grade ≥3 cardiac event incidence when comparing those with prior RT versus without prior RT exceeding LeftTotal dose constraints (A, *P*=0.49). No significant difference in ACE incidence was seen when comparing those with prior RT versus without prior RT that did not exceed LeftTotal dose constraints as well (B, *P*=0.052). No significant differences were seen for proportional hazards between those with prior RT versus without prior RT in both exceeding LeftTotal dose constraints (C) and those that did not exceed constraints (D).

| **Supplemental Table 1.** Individual Coronary Artery Calcium Scores by Visual Scoring | | | | | | |
| --- | --- | --- | --- | --- | --- | --- |
| Pt ID | LMCA | LAD | LCX | RCA | CAC_TotalScore | CAC_TotalGroup |
| 1 | 1 | 1 | 1 | 1 | 4 | Moderate |
| 2 | 0 | 1 | 1 | 2 | 4 | Moderate |
| 3 | 1 | 0 | 1 | 0 | 2 | Mild |
| 4 | 2 | 1 | 0 | 3 | 6 | Moderate |
| 5 | 0 | 0 | 0 | 0 | 0 | None |
| 6 | 1 | 1 | 0 | 1 | 3 | Mild |
| 7 | 1 | 2 | 1 | 2 | 6 | Moderate |
| 8 | 3 | 1 | 3 | 3 | 10 | Severe |
| 9 | 0 | 0 | 0 | 0 | 0 | None |
| 10 | 2 | 1 | 3 | 1 | 7 | Severe |
| 11 | 0 | 0 | 0 | 1 | 1 | Mild |
| 12 | 2 | 2 | 0 | 3 | 7 | Severe |
| 13 | 0 | 1 | 0 | 0 | 1 | Mild |
| 14 | 0 | 2 | 2 | 3 | 7 | Severe |
| 15 | 0 | 0 | 0 | 0 | 0 | None |
| 16 | 0 | 1 | 1 | 1 | 3 | Mild |
| 17 | 3 | 3 | 1 | 1 | 8 | Severe |
| 18 | 1 | 1 | 1 | 0 | 3 | Mild |
| 19 | 0 | 0 | 0 | 0 | 0 | None |
| 20 | 0 | 2 | 2 | 1 | 5 | Moderate |
| 21 | 1 | 1 | 1 | 1 | 4 | Moderate |
| 22 | 0 | 0 | 0 | 0 | 0 | None |
| 23 | 3 | 2 | 1 | 2 | 8 | Severe |
| 24 | 0 | 0 | 0 | 1 | 1 | Mild |
| 25 | 0 | 0 | 0 | 0 | 0 | None |
| 26 | 1 | 1 | 0 | 1 | 3 | Mild |
| 27 | 0 | 1 | 0 | 0 | 1 | Mild |
| 28 | 3 | 1 | 1 | 3 | 8 | Severe |
| 29 | 0 | 0 | 0 | 1 | 1 | Mild |
| 30 | 0 | 3 | 1 | 3 | 7 | Severe |
| 31 | 0 | 1 | 0 | 0 | 1 | Mild |
| 32 | 3 | 1 | 0 | 0 | 4 | Moderate |
| 33 | 0 | 0 | 0 | 1 | 1 | Mild |
| 34 | 0 | 1 | 0 | 0 | 1 | Mild |
| 35 | 0 | 0 | 0 | 0 | 0 | None |
| 36 | 2 | 1 | 3 | 3 | 9 | Severe |
| 37 | 1 | 1 | 0 | 0 | 2 | Mild |
| 38 | 3 | 2 | 1 | 1 | 7 | Severe |
| 39 | 0 | 1 | 0 | 0 | 1 | Mild |
| 40 | 2 | 1 | 1 | 1 | 5 | Moderate |
| 41 | 0 | 1 | 1 | 1 | 3 | Mild |
| 42 | 1 | 1 | 1 | 1 | 4 | Moderate |
| 43 | 2 | 1 | 1 | 0 | 4 | Moderate |
| 44 | 0 | 2 | 1 | 2 | 5 | Moderate |
| 45 | 0 | 0 | 0 | 0 | 0 | None |
| 46 | 3 | 1 | 3 | 3 | 10 | Severe |
| 47 | 3 | 1 | 1 | 3 | 8 | Severe |
| 48 | 1 | 3 | 2 | 3 | 9 | Severe |
| 49 | 0 | 1 | 0 | 0 | 1 | Mild |
| 50 | 0 | 0 | 0 | 0 | 0 | None |
| 51 | 0 | 1 | 0 | 0 | 1 | Mild |
| 52 | 0 | 0 | 0 | 0 | 0 | None |
| 53 | 0 | 0 | 0 | 0 | 0 | None |
| 54 | 0 | 1 | 1 | 0 | 2 | Mild |
| 55 | 0 | 1 | 1 | 2 | 4 | Moderate |
| 56 | 0 | 0 | 0 | 0 | 0 | None |
| 57 | 0 | 2 | 0 | 2 | 4 | Moderate |
| 58 | 0 | 0 | 0 | 0 | 0 | None |
| 59 | 0 | 0 | 0 | 0 | 0 | None |
| 60 | 1 | 2 | 1 | 2 | 6 | Moderate |
| 61 | 0 | 1 | 1 | 0 | 2 | Mild |
| 62 | 3 | 2 | 3 | 1 | 9 | Severe |
| 63 | 3 | 2 | 1 | 2 | 8 | Severe |
| 64 | 0 | 0 | 0 | 0 | 0 | None |
| 65 | 1 | 1 | 2 | 1 | 5 | Moderate |
| 66 | 0 | 0 | 0 | 0 | 0 | None |
| 67 | 0 | 1 | 1 | 1 | 3 | Mild |
| 68 | 2 | 1 | 2 | 1 | 6 | Moderate |
| 69 | 1 | 0 | 0 | 1 | 2 | Mild |
| 70 | 3 | 1 | 1 | 1 | 6 | Moderate |
| 71 | 1 | 0 | 0 | 0 | 1 | Mild |
| 72 | 0 | 1 | 0 | 0 | 1 | Mild |
| 73 | 3 | 1 | 0 | 3 | 7 | Severe |
| 74 | 0 | 0 | 0 | 0 | 0 | None |
| 75 | 0 | 1 | 0 | 0 | 1 | Mild |
| 76 | 0 | 1 | 0 | 0 | 1 | Mild |
| 77 | 0 | 0 | 0 | 0 | 0 | None |
| 78 | 0 | 1 | 0 | 0 | 1 | Mild |
| 79 | 1 | 1 | 0 | 0 | 2 | Mild |
| 80 | 2 | 2 | 1 | 1 | 6 | Moderate |
| 81 | 0 | 0 | 0 | 0 | 0 | None |
| 82 | 0 | 0 | 0 | 0 | 0 | None |
| 83 | 3 | 1 | 0 | 0 | 4 | Moderate |
| 84 | 1 | 0 | 0 | 2 | 3 | Mild |
| 85 | 2 | 1 | 1 | 2 | 6 | Moderate |
| 86 | 3 | 3 | 3 | 1 | 10 | Severe |
| 87 | 3 | 2 | 2 | 3 | 10 | Severe |
| 88 | 1 | 1 | 1 | 1 | 4 | Moderate |
| 89 | 2 | 1 | 1 | 2 | 6 | Moderate |
| 90 | 3 | 1 | 1 | 0 | 5 | Moderate |
| 91 | 1 | 1 | 0 | 0 | 2 | Mild |
| 92 | 3 | 1 | 3 | 0 | 7 | Severe |
| 93 | 3 | 2 | 2 | 1 | 8 | Severe |
| 94 | 0 | 0 | 0 | 0 | 0 | None |
| 95 | 0 | 0 | 0 | 1 | 1 | Mild |
| 96 | 0 | 0 | 0 | 1 | 1 | Mild |
| 97 | 1 | 1 | 1 | 2 | 5 | Moderate |
| 98 | 1 | 1 | 0 | 0 | 2 | Mild |
| 99 | 0 | 1 | 0 | 1 | 2 | Mild |
| 100 | 0 | 0 | 0 | 0 | 0 | None |
| 101 | 0 | 0 | 1 | 3 | 4 | Moderate |
| 102 | 0 | 0 | 0 | 0 | 0 | None |
| 103 | 1 | 2 | 1 | 0 | 4 | Moderate |
| 104 | 1 | 0 | 1 | 1 | 3 | Mild |
| 105 | 0 | 0 | 0 | 0 | 0 | None |
| 106 | 0 | 0 | 0 | 0 | 0 | None |
| 107 | 0 | 0 | 0 | 0 | 0 | None |
| 108 | 0 | 0 | 0 | 0 | 0 | None |
| 109 | 0 | 0 | 0 | 0 | 0 | None |
| 110 | 3 | 1 | 0 | 1 | 5 | Moderate |
| 111 | 0 | 0 | 0 | 0 | 0 | None |
| 112 | 0 | 0 | 0 | 0 | 0 | None |
| 113 | 0 | 0 | 0 | 0 | 0 | None |
| 114 | 1 | 2 | 2 | 3 | 8 | Severe |
| 115 | 0 | 0 | 0 | 0 | 0 | None |
| 116 | 0 | 0 | 0 | 0 | 0 | None |
| 117 | 0 | 0 | 1 | 0 | 1 | Mild |
| 118 | 0 | 1 | 0 | 0 | 1 | Mild |
| 119 | 0 | 0 | 0 | 0 | 0 | None |
| 120 | 1 | 1 | 0 | 0 | 2 | Mild |
| 121 | 0 | 0 | 1 | 2 | 3 | Mild |
| 122 | 0 | 2 | 2 | 2 | 6 | Moderate |
| 123 | 0 | 2 | 2 | 3 | 7 | Severe |
| 124 | 0 | 0 | 0 | 0 | 0 | None |
| 125 | 3 | 1 | 2 | 2 | 8 | Severe |
| 126 | 0 | 0 | 0 | 0 | 0 | None |
| 127 | 3 | 1 | 0 | 2 | 6 | Moderate |
| 128 | 3 | 1 | 2 | 3 | 9 | Severe |
| 129 | 0 | 0 | 0 | 0 | 0 | None |
| 130 | 1 | 0 | 2 | 2 | 5 | Moderate |
| 131 | 0 | 2 | 2 | 1 | 5 | Moderate |
| 132 | 3 | 1 | 1 | 3 | 8 | Severe |
| 133 | 0 | 0 | 0 | 0 | 0 | None |
| 134 | 3 | 1 | 2 | 1 | 7 | Severe |
| 135 | 1 | 1 | 3 | 2 | 7 | Severe |
| 136 | 3 | 3 | 2 | 3 | 11 | Severe |
| 137 | 3 | 2 | 1 | 1 | 7 | Severe |
| 138 | 1 | 1 | 2 | 1 | 5 | Moderate |
| 139 | 3 | 2 | 1 | 1 | 7 | Severe |
| 140 | 0 | 0 | 0 | 0 | 0 | None |
| 141 | 0 | 0 | 1 | 0 | 1 | Mild |
| 142 | 3 | 0 | 0 | 0 | 3 | Mild |
| 143 | 3 | 1 | 1 | 2 | 7 | Severe |
| 144 | 1 | 3 | 3 | 3 | 10 | Severe |
| 145 | 1 | 1 | 1 | 1 | 4 | Moderate |
| 146 | 3 | 1 | 1 | 1 | 6 | Moderate |
| 147 | 3 | 1 | 2 | 3 | 9 | Severe |
| 148 | 0 | 0 | 0 | 1 | 1 | Mild |
| 149 | 1 | 2 | 1 | 2 | 6 | Moderate |
| 150 | 0 | 1 | 0 | 1 | 2 | Mild |
| 151 | 2 | 1 | 1 | 2 | 6 | Moderate |
| 152 | 1 | 2 | 2 | 3 | 8 | Severe |
| 153 | 0 | 2 | 0 | 0 | 2 | Mild |
| 154 | 1 | 1 | 0 | 0 | 2 | Mild |
| 155 | 0 | 0 | 0 | 0 | 0 | None |
| 156 | 3 | 1 | 1 | 1 | 6 | Moderate |
| 157 | 0 | 1 | 0 | 0 | 1 | Mild |
| 158 | 0 | 1 | 1 | 0 | 2 | Mild |
| 159 | 3 | 1 | 1 | 1 | 6 | Moderate |
| 160 | 3 | 3 | 1 | 3 | 10 | Severe |
| 161 | 0 | 0 | 1 | 1 | 2 | Mild |
| 162 | 0 | 0 | 0 | 1 | 1 | Mild |
| 163 | 0 | 1 | 0 | 0 | 1 | Mild |
| 164 | 1 | 2 | 0 | 2 | 5 | Moderate |
| 165 | 3 | 1 | 1 | 3 | 8 | Severe |
| 166 | 3 | 1 | 0 | 3 | 7 | Severe |
| 167 | 1 | 3 | 3 | 1 | 8 | Severe |
| 168 | 2 | 1 | 0 | 1 | 4 | Moderate |
| 169 | 2 | 1 | 1 | 3 | 7 | Severe |
| 170 | 3 | 1 | 2 | 2 | 8 | Severe |
| 171 | 0 | 0 | 1 | 0 | 1 | Mild |
| 172 | 3 | 1 | 1 | 3 | 8 | Severe |
| 173 | 2 | 1 | 0 | 1 | 4 | Moderate |
| 174 | 3 | 1 | 1 | 1 | 6 | Moderate |
| 175 | 1 | 1 | 0 | 0 | 2 | Mild |
| 176 | 3 | 3 | 3 | 3 | 12 | Severe |
| 177 | 0 | 0 | 0 | 0 | 0 | None |
| 178 | 0 | 1 | 0 | 1 | 2 | Mild |
| 179 | 0 | 0 | 0 | 0 | 0 | None |
| 180 | 3 | 3 | 2 | 3 | 11 | Severe |
| 181 | 3 | 2 | 1 | 3 | 9 | Severe |
| 182 | 3 | 2 | 3 | 3 | 11 | Severe |
| 183 | 3 | 3 | 3 | 3 | 12 | Severe |
| 184 | 3 | 0 | 0 | 1 | 4 | Moderate |
| 185 | 3 | 1 | 3 | 3 | 10 | Severe |
| 186 | 0 | 1 | 1 | 2 | 4 | Moderate |
| 187 | 1 | 2 | 2 | 2 | 7 | Severe |
| 188 | 0 | 1 | 1 | 2 | 4 | Moderate |
| 189 | 0 | 0 | 0 | 0 | 0 | None |
| 190 | 1 | 1 | 1 | 1 | 4 | Moderate |
| 191 | 0 | 0 | 0 | 0 | 0 | None |
| 192 | 0 | 0 | 0 | 0 | 0 | None |
| 193 | 1 | 2 | 0 | 1 | 4 | Moderate |
| 194 | 0 | 2 | 2 | 3 | 7 | Severe |
| 195 | 1 | 0 | 0 | 1 | 2 | Mild |
| 196 | 3 | 2 | 1 | 1 | 7 | Severe |
| 197 | 3 | 3 | 2 | 3 | 11 | Severe |
| 198 | 0 | 0 | 0 | 1 | 1 | Mild |
| 199 | 3 | 2 | 1 | 3 | 9 | Severe |
| 200 | 3 | 1 | 0 | 0 | 4 | Moderate |
| 201 | 0 | 0 | 0 | 0 | 0 | None |
| 202 | 1 | 2 | 1 | 1 | 5 | Moderate |
| 203 | 2 | 2 | 0 | 0 | 4 | Moderate |
| 204 | 2 | 2 | 1 | 1 | 6 | Moderate |
| 205 | 3 | 2 | 3 | 3 | 11 | Severe |
| 206 | 0 | 0 | 0 | 0 | 0 | None |
| 207 | 0 | 0 | 0 | 1 | 1 | Mild |
| 208 | 1 | 1 | 0 | 1 | 3 | Mild |
| 209 | 0 | 0 | 1 | 0 | 1 | Mild |
| 210 | 3 | 2 | 2 | 3 | 10 | Severe |
| 211 | 2 | 1 | 2 | 1 | 6 | Moderate |
| 212 | 0 | 0 | 1 | 1 | 2 | Mild |
| 213 | 3 | 2 | 1 | 3 | 9 | Severe |
| 214 | 0 | 0 | 0 | 0 | 0 | None |
| 215 | 0 | 0 | 0 | 0 | 0 | None |
| 216 | 0 | 0 | 0 | 0 | 0 | None |
| 217 | 3 | 1 | 2 | 0 | 6 | Moderate |
| 218 | 2 | 1 | 2 | 1 | 6 | Moderate |
| 219 | 0 | 0 | 0 | 0 | 0 | None |
| 220 | 0 | 0 | 0 | 1 | 1 | Mild |
| 221 | 0 | 1 | 0 | 1 | 2 | Mild |
| 222 | 0 | 0 | 0 | 0 | 0 | None |
| 223 | 3 | 2 | 3 | 3 | 11 | Severe |
| 224 | 3 | 1 | 2 | 3 | 9 | Severe |
| 225 | 2 | 1 | 1 | 1 | 5 | Moderate |
| 226 | 2 | 1 | 3 | 3 | 9 | Severe |
| 227 | 3 | 2 | 3 | 3 | 11 | Severe |
| 228 | 1 | 2 | 0 | 3 | 6 | Moderate |
| 229 | 3 | 1 | 0 | 2 | 6 | Moderate |
| 230 | 0 | 2 | 2 | 0 | 4 | Moderate |
| 231 | 3 | 1 | 2 | 3 | 9 | Severe |
| 232 | 1 | 1 | 1 | 0 | 3 | Mild |
| 233 | 3 | 3 | 2 | 1 | 9 | Severe |
| Legend: CAC = Coronary Artery Calcium Score; LAD = Lower Anterior Descending Artery; LCX = Left Circumflex Artery; LMCA = Left Main Coronary Artery; RCA = Right Coronary Artery | | | | | | |

| **Supplemental Table 2. ROC analyses using time-dependent Cox proportional hazard model for any ACE up to 5 years** | | |
| --- | --- | --- |
| **Metrics** | **AUC** | **95%CI** |
| Mean LCX | 0.59 | 0.46-0.71 |
| Mean TotalLeft | 0.58 | 0.45-0.70 |
| MHD | 0.58 | 0.45-0.70 |
| Mean LAD | 0.57 | 0.45-0.70 |
| Mean LMCA | 0.57 | 0.44-0.69 |
| Mean TotalCor | 0.56 | 0.43-0.69 |
| Mean RCA | 0.49 | 0.36-0.63 |
| Mean LV | 0.63 | 0.52-0.75 |
| Mean RA | 0.57 | 0.47-0.67 |
| CAC LCX | 0.64 | 0.52-0.75 |
| CAC TotalLeft | 0.62 | 0.49-0.74 |
| CAC LMCA | 0.60 | 0.49-0.71 |
| CAC LAD | 0.56 | 0.44-0.68 |
| CAC RCA | 0.58 | 0.47-0.69 |
| CAC TotalCor | 0.61 | 0.49-0.73 |
| LV V15+CAC | 0.65 | 0.55-0.75 |
| TotalLeft V15+CAC | 0.69 | 0.59-0.80 |
| LAD V15+CAC | 0.67 | 0.56-0.77 |
| LCX V15+CAC | 0.67 | 0.56-0.78 |
| LMCA V15+CAC | 0.64 | 0.52-0.75 |
| MHD+CAC | 0.64 | 0.54-0.75 |
| Abbreviations: ACE=Grade ≥3 Adverse Cardiac Events, AUC=Area Under Curve, CAC=Coronary Artery Calcium score, LAD=left anterior descending artery, LCX=left circumflex artery, LMCA=left main coronary artery, MHD=mean heart dose, LeftTotal=LAD+LCX+LMCA, RCA=Right Coronary Artery, ROC=Receiver Operating Characteristic, TotalCor=LAD+LCX+LMCA+RCA, V15=Volume receiving 15 Gray | | |

| **Supplemental Table 3.** Individual Systemic Therapy Courses | | | | |
| --- | --- | --- | --- | --- |
| Pt ID | chemo_list | immuno | targeted | chemo_cycles |
| 1 | carboplatin/paclitaxel | 0 | 0 | 4 |
| 2 | carboplatin/paclitaxel | 0 | 0 | 6 |
| 3 | carboplatin/paclitaxel | 0 | 0 | 6 |
| 4 | carboplatin/paclitaxel | 0 | 0 | 6 |
| 5 | carboplatin/paclitaxel | 0 | 0 | 6 |
| 6 | carboplatin/paclitaxel | 0 | 0 | 3 |
| 7 | carboplatin/paclitaxel | 0 | 0 | 6 |
| 8 | unknown | 0 | 0 | unknown |
| 9 | carbo/taxol discontinued cycle 3; 2/2 taxol reaction and switched to weekly abraxane | 0 | 0 | unknown |
| 10 | carboplatin/paclitaxel | 0 | 0 | 1 |
| 11 | carboplatin/paclitaxel | 0 | 0 | 6 |
| 12 | carboplatin/paclitaxel | 0 | 0 | 5 |
| 13 | carboplatin/pemetrexed | 0 | 0 | 5 |
| 14 | cisplatin/pemetrexed | 0 | 0 | unknown |
| 15 | carboplatin/pemetrexed | 0 | 0 | 4 |
| 16 | carboplatin/pemetrexed | 0 | 0 | 4 |
| 17 | carboplatin/pemetrexed | 0 | 0 | 3 |
| 18 | carboplatin/paclitaxel | 0 | 0 | 4 |
| 19 | carboplatin/paclitaxel | 0 | 0 | 5 |
| 20 | carboplatin/paclitaxel | 0 | 0 | 5 |
| 21 | carboplatin/paclitaxel | 0 | 0 | 6 |
| 22 | carboplatin/pemetrexed | 0 | 0 | 2 |
| 23 | carboplatin/paclitaxel | 0 | 0 | 1 |
| 24 | carboplatin/paclitaxel | 0 | 0 | 4 |
| 25 | carboplatin/paclitaxel | 0 | 0 | 5 |
| 26 |  | 0 | 0 |  |
| 27 | cisplatin/pemetrexed | 0 | 0 | 1 |
| 28 | carboplatin/paclitaxel | 0 | 0 | 6 |
| 29 | carboplatin/paclitaxel | 0 | 0 | 7 |
| 30 | carboplatin/paclitaxel | 0 | 0 | 5 |
| 31 | cisplatin/pemetrexed | 0 | 0 | 2 |
| 32 | carboplatin/paclitaxel | 0 | 0 | 7 |
| 33 | carboplatin/paclitaxel | 0 | 0 | 5 |
| 34 | carboplatin/paclitaxel | 0 | 0 | 6 |
| 35 | carboplatin/paclitaxel | 0 | 0 | 5 |
| 36 | carboplatin/paclitaxel | 0 | 0 | 3 |
| 37 | carboplatin/paclitaxel | 0 | 0 | 5 |
| 38 | carboplatin/paclitaxel | 0 | 0 | 6 |
| 39 | carboplatin/paclitaxel | 0 | 0 | 7 |
| 40 | carboplatin/paclitaxel | 0 | 0 | 3 |
| 41 | carboplatin/paclitaxel | 0 | 0 | 5 |
| 42 | carboplatin/paclitaxel | 0 | 0 | 6 |
| 43 | carboplatin/paclitaxel | 0 | 0 | 6 |
| 44 | carboplatin/paclitaxel | 0 | 0 | 6 |
| 45 | carboplatin/paclitaxel | 0 | 0 | 5 |
| 46 | carboplatin/paclitaxel | 0 | 0 | 6 |
| 47 | carboplatin/paclitaxel | 0 | 0 | 6 |
| 48 | carboplatin/paclitaxel | 0 | 0 | 5 |
| 49 | carboplatin/paclitaxel | 0 | 0 | 7 |
| 50 | carboplatin/paclitaxel | 0 | 0 | 3 |
| 51 | carboplatin/paclitaxel | 0 | 0 | 5 |
| 52 | cisplatin/etoposide | 0 | 0 | 2 |
| 53 | carboplatin/paclitaxel | 0 | 0 | 6 |
| 54 |  | 0 | 0 |  |
| 55 | cisplatin/etoposide (1) then switched to carboplatin/paclitaxel (2) | 0 | 0 | 3 |
| 56 | carboplatin/pemetrexed | 0 | 0 | 3 |
| 57 | carboplatin/pemetrexed | 0 | 0 | 2 |
| 58 | cisplatin/pemetrexed | 0 | 0 | 3 |
| 59 | cisplatin/pemetrexed | 0 | 0 | 3 |
| 60 | carboplatin/paclitaxel | 0 | 0 | 6 |
| 61 | carboplatin/pemetrexed | 0 | 0 | 4 |
| 62 | carboplatin/pemetrexed | 0 | 0 | 3 |
| 63 | carboplatin/paclitaxel | 0 | 0 | 7 |
| 64 | cisplatin/etoposide | 0 | 0 | 2 |
| 65 | carboplatin/pemetrexed | 0 | 0 | 2 |
| 66 | carboplatin/paclitaxel | 0 | 0 | 7 |
| 67 | carboplatin/paclitaxel | 0 | 0 | 4 |
| 68 | carboplatin/paclitaxel | 0 | 0 | 7 |
| 69 | carboplatin/paclitaxel | 0 | 0 | 6 |
| 70 | cisplatin/etoposide | 0 | 0 | 2 |
| 71 | carboplatin/paclitaxel | 0 | 0 | 6 |
| 72 | carboplatin/paclitaxel | 0 | 0 | 6 |
| 73 | carboplatin/abraxane | 0 | 0 | unknown |
| 74 | carboplatin/paclitaxel | 0 | 0 | 6 |
| 75 | carboplatin/paclitaxel | 0 | 0 | 6 |
| 76 | carboplatin/paclitaxel | 0 | 0 | 6 |
| 77 | carboplatin/paclitaxel | 0 | 0 | 5 |
| 78 | carboplatin/paclitaxel + panitumumab | 1 | 0 | unknown |
| 79 | carboplatin/paclitaxel | 0 | 0 | 3 |
| 80 | carboplatin/paclitaxel | 0 | 0 | 5 |
| 81 | carboplatin/paclitaxel | 0 | 0 | 7 |
| 82 | carboplatin/paclitaxel | 0 | 0 | 6 |
| 83 | carboplatin/paclitaxel | 0 | 0 | 6 |
| 84 | carboplatin/paclitaxel | 0 | 0 | 6 |
| 85 | carboplatin/paclitaxel | 0 | 0 | 6 |
| 86 | carboplatin/paclitaxel | 0 | 0 | 6 |
| 87 | carboplatin/paclitaxel | 0 | 0 | 10 |
| 88 | carboplatin/paclitaxel | 0 | 0 | 6 |
| 89 | carboplatin/paclitaxel | 0 | 0 | 8 |
| 90 | carboplatin/paclitaxel | 0 | 0 | 5 |
| 91 | carboplatin/paclitaxel | 0 | 0 | 6 |
| 92 | carboplatin/paclitaxel | 0 | 0 | 3 |
| 93 | carboplatin/paclitaxel | 0 | 0 | 1 |
| 94 | carboplatin/paclitaxel | 0 | 0 | 6 |
| 95 | carboplatin/paclitaxel | 0 | 0 | 5 |
| 96 | carboplatin/paclitaxel/erbitux | 1 | 0 | unknown |
| 97 | carboplatin/pemetrexed | 0 | 0 | 3 |
| 98 | carboplatin/pemetrexed | 0 | 0 | 2 |
| 99 | carboplatin/pemetrexed | 0 | 0 | unknown |
| 100 | carboplatin/pemetrexed | 0 | 0 | 3 |
| 101 | carboplatin/pemetrexed | 0 | 0 | 2 |
| 102 | cisplatin/etoposide | 0 | 0 | 4 |
| 103 | etoposide | 0 | 0 | 4 |
| 104 | cisplatin/etoposide | 0 | 0 | 4 |
| 105 | cisplatin/etoposide | 0 | 0 | 2 |
| 106 | cisplatin/etoposide | 0 | 0 | 2 |
| 107 | cisplatin/etoposide | 0 | 0 | 2 |
| 108 | cisplatin/etoposide | 0 | 0 | 2 |
| 109 | cisplatin/etoposide | 0 | 0 | 4 |
| 110 | cisplatin/etoposide | 0 | 0 | 2 |
| 111 | cisplatin/etoposide | 0 | 0 | 2 |
| 112 | carboplatin/pemetrexed | 0 | 0 | 2 |
| 113 | carboplatin/pemetrexed | 0 | 0 | 4 |
| 114 | carboplatin/pemetrexed | 0 | 0 | 2 |
| 115 | carboplatin/pemetrexed | 0 | 0 | 4 |
| 116 | carboplatin/pemetrexed | 0 | 0 | 4 |
| 117 | carboplatin/pemetrexed | 0 | 0 | 5 |
| 118 | carboplatin/pemetrexed | 0 | 0 | 6 |
| 119 | carboplatin/pemetrexed | 0 | 0 | 12 |
| 120 | carboplatin/paclitaxel | 0 | 0 | 5 |
| 121 | cisplatin/etoposide | 0 | 0 | 2 |
| 122 | carboplatin/paclitaxel | 0 | 0 | 1 |
| 123 | cisplatin/etoposide | 0 | 0 | 3 |
| 124 | carboplatin/etoposide | 0 | 0 | 6 |
| 125 | carboplatin/paclitaxel | 0 | 0 | 8 |
| 126 | cisplatin/etoposide | 0 | 0 | 3 |
| 127 | carboplatin/paclitaxel | 0 | 0 | 6 |
| 128 | carboplatin/paclitaxel | 0 | 0 | 6 |
| 129 | carboplatin/paclitaxel | 0 | 0 | 6 |
| 130 | carboplatin/paclitaxel | 0 | 0 | 5 |
| 131 | carboplatin/paclitaxel | 0 | 0 | 3 |
| 132 | carboplatin/paclitaxel | 0 | 0 | 6 |
| 133 | carboplatin/paclitaxel | 0 | 0 | 5 |
| 134 | carboplatin/paclitaxel | 0 | 0 | 6 |
| 135 | carboplatin/paclitaxel | 0 | 0 | 1 |
| 136 | carboplatin/paclitaxel | 0 | 0 | 6 |
| 137 | carboplatin/paclitaxel | 0 | 0 | 6 |
| 138 | carboplatin/paclitaxel | 0 | 0 | 8 |
| 139 | carboplatin/paclitaxel | 0 | 0 | 6 |
| 140 | carboplatin/paclitaxel for 2 cycles then switched to cisplatin/etoposide (unknown # cycles) | 0 | 0 | unknown |
| 141 | cisplatin/etoposide | 0 | 0 | 2 |
| 142 | carboplatin/paclitaxel | 0 | 0 | 5 |
| 143 | carboplatin/paclitaxel | 0 | 0 | 7 |
| 144 | carboplatin/paclitaxel | 0 | 0 | 6 |
| 145 | carboplatin/paclitaxel | 0 | 0 | 7 |
| 146 | cisplatin | 0 | 0 | unknown |
| 147 | carboplatin/paclitaxel | 0 | 0 | 5 |
| 148 | carboplatin/paclitaxel | 0 | 0 | 7 |
| 149 | carboplatin/paclitaxel | 0 | 0 | 6 |
| 150 | carboplatin/paclitaxel | 0 | 0 | 5 |
| 151 | carboplatin/paclitaxel | 0 | 0 | 6 |
| 152 | carboplatin/paclitaxel | 0 | 0 | 7 |
| 153 | carboplatin/paclitaxel | 0 | 0 | 8 |
| 154 | carboplatin/paclitaxel | 0 | 0 | 5 |
| 155 | carboplatin/paclitaxel | 0 | 0 | 8 |
| 156 | carboplatin/paclitaxel | 0 | 0 | 5 |
| 157 | Gemzar, carboplatin, and Avastin | 0 | 1 | 7 |
| 158 | carboplatin/paclitaxel | 0 | 0 | 7 |
| 159 | carboplatin/paclitaxel | 0 | 0 | 6 |
| 160 | carboplatin/paclitaxel | 0 | 0 | 5 |
| 161 | cisplatin/pemetrexed | 0 | 0 | 3 |
| 162 | carboplatin/paclitaxel | 0 | 0 | 6 |
| 163 | carboplatin/paclitaxel | 0 | 0 | 8 |
| 164 | cisplatin/etoposide | 0 | 0 | 2 |
| 165 | carboplatin/paclitaxel | 0 | 0 | 5 |
| 166 | carboplatin/paclitaxel | 0 | 0 | 6 |
| 167 | carboplatin/paclitaxel | 0 | 0 | 5 |
| 168 | carboplatin/paclitaxel | 0 | 0 | 6 |
| 169 | carboplatin/paclitaxel | 0 | 0 | 5 |
| 170 | carboplatin/paclitaxel | 0 | 0 | 5 |
| 171 | crizotinib | 0 | 1 | unknown |
| 172 | carboplatin/paclitaxel | 0 | 0 | 4 |
| 173 | carboplatin/paclitaxel | 0 | 0 | 3 |
| 174 |  | 0 | 0 |  |
| 175 | carboplatin/paclitaxel | 0 | 0 | 6 |
| 176 | carboplatin/paclitaxel | 0 | 0 | 8 |
| 177 | carboplatin/paclitaxel | 0 | 0 | 7 |
| 178 | carboplatin/paclitaxel | 0 | 0 | 6 |
| 179 | carboplatin/paclitaxel | 0 | 0 | 6 |
| 180 | carboplatin/paclitaxel | 0 | 0 | 3 |
| 181 | carboplatin/paclitaxel | 0 | 0 | 7 |
| 182 | carboplatin/paclitaxel | 0 | 0 | 6 |
| 183 | carboplatin/paclitaxel | 0 | 0 | 4 |
| 184 | carboplatin/paclitaxel | 0 | 0 | 6 |
| 185 | carboplatin/paclitaxel | 0 | 0 | 6 |
| 186 | carboplatin/paclitaxel | 0 | 0 | 4 |
| 187 | carboplatin/paclitaxel | 0 | 0 | 5 |
| 188 | carboplatin/paclitaxel | 0 | 0 | 2 |
| 189 | cisplatin/etoposide | 0 | 0 | 2 |
| 190 | carboplatin/paclitaxel | 0 | 0 | 6 |
| 191 | carboplatin/paclitaxel | 0 | 0 | 3 |
| 192 | carboplatin/paclitaxel | 0 | 0 | 6 |
| 193 | carboplatin/paclitaxel | 0 | 0 | 6 |
| 194 | carboplatin/pemetrexed | 0 | 0 | 3 |
| 195 | cisplatin/etoposide | 0 | 0 | 2 |
| 196 |  | 0 | 0 |  |
| 197 | carboplatin/paclitaxel | 0 | 0 | 6 |
| 198 | carboplatin/paclitaxel | 0 | 0 | 6 |
| 199 | carboplatin/paclitaxel | 0 | 0 | 5 |
| 200 | carboplatin/paclitaxel | 0 | 0 | 8 |
| 201 | carboplatin/paclitaxel | 0 | 0 | 6 |
| 202 | carboplatin/paclitaxel | 0 | 0 | 6 |
| 203 | cisplatin/etoposide | 0 | 0 | 2 |
| 204 | carboplatin/paclitaxel | 0 | 0 | 4 |
| 205 | carboplatin | 0 | 0 | 4 |
| 206 | cisplatin/etoposide | 0 | 0 | 2 |
| 207 | cisplatin/pemetrexed | 0 | 0 | 4 |
| 208 | carboplatin/paclitaxel | 0 | 0 | 6 |
| 209 | carboplatin/pemetrexed | 0 | 0 | 2 |
| 210 | carboplatin/paclitaxel | 0 | 0 | 6 |
| 211 | carboplatin/paclitaxel | 0 | 0 | 6 |
| 212 | carboplatin/paclitaxel | 0 | 0 | 3 |
| 213 | carboplatin/paclitaxel | 0 | 0 | 6 |
| 214 | cisplatin/etoposide | 0 | 0 | 2 |
| 215 | carboplatin/paclitaxel | 0 | 0 | 6 |
| 216 | taxol (low dose) | 0 | 0 | 2 |
| 217 | carboplatin/paclitaxel | 0 | 0 | 6 |
| 218 | carboplatin/pemetrexed | 0 | 0 | 6 |
| 219 | carboplatin/pemetrexed | 0 | 0 | 4 |
| 220 | cisplatin/etoposide | 0 | 0 | 2 |
| 221 | cisplatin/etoposide | 0 | 0 | 3 |
| 222 | cisplatin/etoposide | 0 | 0 | 2 |
| 223 | carboplatin/paclitaxel | 0 | 0 | 6 |
| 224 | carboplatin/paclitaxel | 0 | 0 | 4 |
| 225 | carboplatin/paclitaxel | 0 | 0 | 4 |
| 226 | cisplatin/etoposide | 0 | 0 | 3 |
| 227 | carboplatin/paclitaxel | 0 | 0 | 6 |
| 228 |  | 0 | 0 |  |
| 229 | carboplatin/paclitaxel | 0 | 0 | 5 |
| 230 | carboplatin/paclitaxel | 0 | 0 | 7 |
| 231 | carboplatin/paclitaxel | 0 | 0 | 6 |
| 232 | carboplatin/paclitaxel | 0 | 0 | 7 |
| 233 | carboplatin/paclitaxel | 0 | 0 | 8 |
